# Therapie der chronischen Rhinosinusitis mit Polyposis nasi (CRScNP) mit monoklonalen Antikörpern (Biologika): S2k-Leitlinie der Deutschen Gesellschaft für Hals-Nasen-Ohren-Heilkunde, Kopf- und Hals-Chirurgie (DGHNO-KHC) und der Deutschen Gesellschaft für Allgemeinmedizin und Familienmedizin (DEGAM)

**DOI:** 10.1007/s00106-023-01273-2

**Published:** 2023-03-20

**Authors:** Oliver Pfaar, Achim Georg Beule, Martin Laudien, Boris A. Stuck, erweiterte Leitliniengruppe ‚Biologika bei CRScNP‘, Christoph Aletsee, Ludger Klimek, Katrin Milger-Kneidinger, Uwe Popert, Markus Rose, Martin Wagenmann

**Affiliations:** 1 Klinik für Hals‑, Nasen- und Ohrenheilkunde, Kopf- und Hals-Chirurgie, Universitätsklinikum Gießen und Marburg GmbH, Philipps-Universität Marburg, Baldingerstraße, 35043 Marburg, Deutschland; 2 Klinik für Hals‑, Nasen- und Ohrenheilkunde, Universitätsklinikum Münster, Münster, Deutschland; 3 Klinik und Poliklinik für Hals-Nasen-Ohrenheilkunde, Kopf- und Halschirurgie, Universitätsmedizin Greifswald, Greifswald, Deutschland; 4 Klinik für HNO-Heilkunde, Kopf- und Halschirurgie, Universitätsklinikum Schleswig-Holstein, Campus Kiel, Christian-Albrechts-Universität zu Kiel, Kiel, Deutschland

**Keywords:** Sinusitis, Nasennebenhöhlenerkrankungen, Asthma, Nasenpolypen, Sinusitis, Sinunasal diseases, Asthma, Nasal polyps

## Abstract

Monoklonale Antikörper (sog. Biologika) können bei chronischer Rhinosinusitis mit Nasenpolypen (CRScNP, im englischen Sprachgebrauch CRSwNP) im Rahmen ihrer Zulassung verordnet werden. Allerdings ist die Verordnung auf die schwere CRScNP ohne Krankheitskontrolle begrenzt, wobei bestimmte Voraussetzungen zu beachten sind. Derzeit sind Dupilumab, Omalizumab und Mepolizumab zugelassen, in der Literatur gibt es entsprechende Evidenz für ihre Wirksamkeit und Sicherheit. Es ist davon auszugehen, dass zukünftig weitere Biologika in dieser Indikation zugelassen werden. Die Schwere der Erkrankung sollte vor Therapieeinleitung sowie die Wirksamkeit dieser Therapie nach einem angemessenen Zeitraum objektiv und subjektiv überprüft werden. Hierfür kann der in diesem aktualisierten Leitlinien-Kapitel vorgeschlagene Dokumentationsbogen verwendet werden. Bei Vorliegen von relativen Kontraindikationen sollte nur nach differenzierter Abwägung durch erfahrene Ärzt*innen und als Einzelfallentscheidung ein Therapieversuch mit Biologika erfolgen. Zusammenfassend hat das vorliegende Leitlinien-Kapitel zum Ziel, angesichts der zunehmenden Evidenz zur Therapie mit diesen Substanzen bzw. der zunehmenden Zahl an Zulassungen unterschiedlicher Biologika zu einer qualitativ hochwertigen Versorgung von erwachsenen Patient*innen mit dieser Therapieform beizutragen.

**Zusatzmaterial online:**

Die Online-Version dieses Beitrags (10.1007/s00106-023-01273-2) enthält weitere Tabellen/Texte: 1: Systematische Bewertung der Evidenz, 2: Leitlinienreport, 3: Dokumentationsbogen.

## Abstract (en)

Monoclonal antibodies (so-called biologics) can be prescribed for chronic rhinosinusitis with nasal polyps (CRSwNP) within the scope of their market authorization. However, their prescription is limited to severe CRSwNP without disease control, whereby certain requirements must be met. Dupilumab, omalizumab, and mepolizumab have currently gained market authorization, with adequate evidence for their efficacy and safety available in the literature. It can be assumed that other biologics will be approved for this indication in the future. The severity of disease and the efficacy of treatment should be assessed objectively and subjectively before treatment initiation and after an appropriate duration, respectively. The documentation sheet proposed in this guideline chapter can be used for the assessments. In the presence of relative contraindications, a treatment should only be initiated after differentiated consideration by an experienced physician in the sense of a case-by-case decision. In summary, this guideline chapter aims to contribute to high-quality care of adult patients with these therapies in view of the increasing evidence for treatment with these substances and the increasing number of market authorizations of different biologics.

## Mitglieder erweiterte Leitliniengruppe ***Biologika bei CRScNP***

Christoph Aletsee (HNO-Gemeinschaftspraxis, Bad Kreuznach); Ludger Klimek (Allergiezentrum Wiesbaden); Katrin Milger-Kneidinger (Medizinische Klinik und Poliklinik V, LMU Klinikum, Campus Großhadern, München); Uwe Popert (Praxis für Allgemeinmedizin, Kassel); Markus Rose (Pädiatrische Pneumologie, Allergologie und CF-Zentrum, Klinikum Stuttgart, Stuttgart); Martin Wagenmann (HNO-Klinik, Universitätsklinikum Düsseldorf)

## Infobox

AWMF-Leitlinien Registernummer 017-049: S2k-Leitlinie Rhinosinusitis

Federführend herausgegeben von: Deutsche Gesellschaft für Hals-Nasen-Ohren-Heilkunde, Kopf- und Hals-Chirurgie e. V. (DGHNO-KHC): Deutsche Gesellschaft für Allgemeinmedizin und Familienmedizin e. V. (DEGAM)

Verantwortlich für die Überarbeitung:

Prof. Dr. med. Boris A. Stuck (DGHNO),

Dr. med. Uwe Popert (DEGAM)

https://register.awmf.org/de/leitlinien/detail/017-049

## 1. Ziele, Zielgruppen und Geltungsbereich der Leitlinie

### 1.1 Ziele und Zielgruppe

Monoklonale Antikörper (sog. Biologika) können bei der chronischen Rhinosinusitis mit Nasenpolypen (CRScNP, im englischen Sprachgebrauch CRSwNP) im Rahmen der Zulassung dieser Substanzen verordnet und angewendet werden.

Das vorliegende Leitlinien(LL)-Kapitel hat zum Ziel, angesichts der zunehmenden Evidenz zur Therapie mit diesen Substanzen bzw. der zunehmenden Zahl an Zulassungen unterschiedlicher Biologika zu einer qualitativ hochwertigen Versorgung von erwachsenen Patient*innen mit dieser Therapieform beizutragen. Es soll über die indikationsgerechte Diagnostik und Kriterien für den Einsatz von Biologika bei CRScNP informieren mit dem Ziel, die krankheitsbedingte Morbidität zu mindern. Darüber hinaus hat das LL-Kapitel zum Ziel, vor dem Hintergrund der derzeit erheblichen Therapiekosten unter sozioökonomischen Gesichtspunkten einen optimalen Einsatz dieser Biologika zu gewährleisten.

### 1.2 Geltungsbereich

Das folgende Kapitel ist für die Versorgung von Patient*innen mit CRScNP im primär- und sekundärärztlichen Bereich vorgesehen. Primärärzt*innen verantworten die Erstbehandlung der Patient*innen, wobei diese Funktion in erster Linie unabhängig ist vom ärztlichen Fachgebiet bzw. der ärztlichen Spezialisierung. Sekundärärzt*innen sind in die Weiterbehandlung der Patient*innen eingebunden und behandeln die Patient*innen nach Überweisung durch den/die Primärärzt*in.

## 2. Nationale und internationale Leitlinien

Leitlinien sind systematisch entwickelte Empfehlungen, die Grundlagen für die gemeinsame Entscheidung von Ärzt*innen und deren Patient*innen zu einer im Einzelfall sinnvollen gesundheitlichen Versorgung darstellen.

Für das vorliegende Kapitel erfolgte eine systematische Aufbereitung der Evidenz (ESM 1, Tabelle S1/S2/S3), sodass der erste Schritt zur Weiterentwicklung auf eine S3-Leitlinie erfolgt ist. Für die anstehende Aktualisierung der Gesamtleitlinie ist eine Weiterentwicklung auf S3-Niveau für die gesamte Leitlinie geplant. Da die Leitlinie formal vonseiten der AWMF auf die Kriterien einer S2k-Leitlinie geprüft wurde, wurde auf die Vergabe von Evidenz- und Empfehlungsgraden verzichtet. Weiterführende Details zur LL-Erstellung finden sich im LL-Report (ESM 2). Das aktualisierte LL-Kapitel basiert auf der im Jahr 2017 publizierten gemeinsamen LL der Deutschen Gesellschaft für Hals-Nasen-Ohren-Heilkunde, Kopf- und Hals-Chirurgie (DGHNO-KHC) und der der Deutschen Gesellschaft für Allgemeinmedizin und Familienmedizin (DEGAM) [1].

Für die nun vorliegende gemeinsame Teilaktualisierung zur Therapie mit Biologika wurden bis 11/2020 aktuelle von den LL-Autoren gefundene nationale und internationale LL und Positionspapiere zur CRS berücksichtigt. Hierbei wurde zudem auf eine kürzlich publizierte systematische Analyse der vorhandenen Leitlinien zurückgegriffen [2]. Von ausreichender methodischer Qualität und Aktualität waren hierbei die folgenden Publikationen:

- die S2k-Leitlinie „*Rhinosinusitis“* der DGHNO-KHC und DEGAM von 2017 [1],
- das „*European Position Paper on Rhinosinusitis and Nasal Polyps*“ (EPOS) in der Aktualisierung aus dem Jahr 2020 [3],
- das Experten-Konsensuspapier „*EUFOREA expert board meeting on uncontrolled severe chronic rhinosinusitis with nasal polyps (CRSwNP) and biologics: Definitions and management*“ [4],
- das Positionspapier „*Anwendung von Biologika bei chronischer Rhinosinusitis mit Polyposis nasi (CRSwNP) im deutschen Gesundheitssystem – Empfehlungen des Ärzteverbandes Deutscher Allergologen (AeDA) und der AGs Klinische Immunologie, Allergologie und Umweltmedizin und Rhinologie und Rhinochirurgie der Deutschen Gesellschaft für HNO-Heilkunde, Kopf- und Halschirurgie (DGHNO-KHC)*“ [5].

Zusätzlich zu den genannten LL und Positionspapieren wurde eine systematische Literaturrecherche zur Therapie der CRScNP des Erwachsenen mit Biologika durchgeführt. Details zur Literaturrecherche (ESM 1, Tabelle S1/S2/S3) können dem LL-Protokoll (ESM 2) entnommen werden.

## 3. Monoklonale Antikörper/Biologika in klinischer Entwicklung und/oder Zulassung: Übersicht über die Evidenz

### 3.1 Dupilumab

Dupilumab ist ein humaner monoklonaler Antikörper. Er hemmt den IL4-/IL-13-Signalweg durch Bindung an die Alpha-Untereinheit des IL-4-Rezeptors [6, 7]. Dupilumab ist seit Oktober 2019 zur Behandlung der schweren CRScNP bei fortgesetzter Therapie mit intranasalen Steroiden (INS) zugelassen (Fach- und Gebrauchsinformation [8]). Zudem liegt für dieses Präparat eine umfangreiche Nutzenbewertung des Instituts für Qualität und Wirtschaftlichkeit im Gesundheitswesen (IQWiG) vor [9].

Es konnten 4 Publikationen zu 3 RCT-Studien zur Beurteilung der Effekte einer Therapie mit Dupilumab detektiert werden (Tabelle S1; [6, 10–13]). Die Dosierungen lagen bei 300 mg wöchentlich bis 2‑wöchentlich subkutan (s.c.) mit/ohne erhöhte Startdosis von 600 mg [8]. Die Effekte wurden nach 16–52 Wochen Therapie analysiert. Die eingeschlossenen Patient*innen waren im Mittel 50 Jahre alt und zeigten Symptome trotz Standardtherapie (INS, oGKS oder Operationen der Nasennebenhöhlen). In den mit Dupilumab behandelten Gruppen lag bei 16–63 % der Patient*innen ein komorbides Asthma vor. Primäre und sekundäre Endpunkte der Studien waren: Nasal Polyp Score (NPS), Nasal Congestion Score (NCS), Lund-Mackay-Score (LMK), nach Zinreich modifizierter LMK (zLMK), Sino-Nasal Outcome Test-22 (SNOT-22), University of Pennsylvania Smell Identification Test (UPSIT), Visuelle Analogskalen der Schwere der Symptome der Erkrankung (VAS) und gesundheitsbezogene Lebensqualität (Health-related Quality of Life, HrQoL). Für alle untersuchten Parameter ergaben sich, meist schon nach kurzer Therapiedauer, im Vergleich zu den mit Placebo behandelten Gruppen signifikante Verbesserungen unter Therapie. Allein die VAS zeigte in einer der Studien einen Trend zu positiver Wirkung, ohne Signifikanzniveau zu erreichen [12]. Subgruppen der CRScNP hinsichtlich der Effekte lassen sich in den vorliegenden Arbeiten nicht detektieren bzw. sind in den vorliegenden Publikationen nicht ausgewiesen.

### 3.2 Omalizumab

Omalizumab ist ein rekombinanter, humanisierter monoklonaler Antikörper. Omalizumab bindet an Immunglobulin E (IgE) und verhindert damit die Bindung von IgE an den FcεRI (IgE-Rezeptor auf Mastzellen und Basophilen) [6, 7]. Omalizumab ist seit August 2020 als Zusatztherapie zu INS zur Behandlung der schweren CRScNP zugelassen (Fach- und Gebrauchsinformation [14]).

Es konnten 4 RCT-Studien [15–17] und 3 Fall-Kontroll-Studien [18–20] zur Beurteilung der Effekte einer Therapie mit Omalizumab detektiert werden (Tabelle S1). Die Dosierungen lagen bei 75–600 mg (nach Körpergewicht und IgE-Basiswert) 2‑ oder 4‑wöchentlich s.c. Die Effekte wurden nach 16–52 Wochen Therapie analysiert. Die eingeschlossenen Patient*innen waren im Mittel zwischen 28 und 52 Jahre alt und zeigten Symptome trotz Standardtherapie (INS, oGKS oder Operationen der Nasennebenhöhlen). In den mit Omalizumab behandelten Gruppen lag in 5 der 7 Studien [15, 16, 19, 20] bei allen Patient*innen eine CRScNP vor, in den weiteren 2 Studien fand sich bei nur 86 % bzw. 32 % der eingeschlossenen Patient*innen eine Polyposis nasi [17, 18]. Der Anteil an Patient*innen mit komorbidem (allergischem) Asthma und Sensibilisierung auf inhalative Allergene variierte in den Studien.

Primäre und sekundäre Endpunkte der Studien waren: NPS, Änderung der (Ko‑)Medikation, SNOT-22, LMK, subjektive Einschätzung der Symptomenschwere, HRQoL, der Peak Inspiratory Nasal Flow (PNIF), UPSIT und Zelluntersuchung in der nasalen Lavage. Für die überwiegende Zahl der untersuchten Parameter ergaben sich im Vergleich zu den mit Placebo behandelten Gruppen signifikante Verbesserungen unter Therapie meist schon nach kurzer Therapiedauer. Subgruppen der CRScNP hinsichtlich der Effekte lassen sich in den vorliegenden Arbeiten nicht detektieren bzw. sind in den vorliegenden Publikationen nicht ausgewiesen.

### 3.3 Mepolizumab

Mepolizumab ist ein humanisierter monoklonaler Antikörper (IgG1, Kappa), der an humanes Interleukin‑5 (IL-5) bindet [6, 7]. Mepolizumab ist seit November 2021 als Zusatztherapie zu intranasaler Kortikoidmedikation zur Behandlung der CRScNP zugelassen (Fach- und Gebrauchsinformation [21]).

Es konnten 3 RCT-Studien [22–24] und eine Fall-Kontroll-Studie [25] zur Beurteilung der Effekte einer Therapie detektiert werden (Tabelle S1). Die Dosierungen und Verabreichungswege unterschieden sich in den Studien erheblich mit Dosierungen zwischen 100 und 750 mg alle 4 Wochen i.v. oder s.c. Die Effekte wurden nach 8–52 Wochen Therapie analysiert. Die eingeschlossenen Patient*innen waren etwa 50 Jahre alt und zeigten Symptome trotz Standardtherapie (INS, oGKS oder Operationen der Nasennebenhöhlen). Bis zu 100 % der eingeschlossenen Patient*innen hatten ein komorbides Asthma, eine vorausgegangene Operation der Nasennebenhöhlen und/oder eine Analgetikaintoleranz. Primäre und sekundäre Endpunkte der Studien waren: NPS, NCS, Änderung der Basismedikation, Änderung der OP-Indikation, VAS, SNOT-22, HrQoL, PNIF, Sniffin’ Sticks, Blut- und Serummarker, Biomarker im nasalen Sekret, Veränderungen in der Computertomographie (CT), Notwendigkeit einer Operation oder der Gabe von oGKS. Für die meisten untersuchten Parameter ergaben sich im Vergleich zu den mit Placebo behandelten Gruppen signifikante Verbesserungen unter Therapie, meist schon nach kurzer Therapiedauer. Kriterien zur Differenzierung von Respondern/Nonrespondern waren nicht zu detektieren [23].

### 3.4 Reslizumab

Reslizumab ist ein monoklonaler Anti-Interleukin(IL-)5-Antikörper [6, 7]. Reslizumab ist für die Indikation CRScNP nicht zugelassen (Stand November 2022). Eine RCT-Studie konnte zur Beurteilung der Effekte einer Behandlung der CRScNP detektiert werden ([26]; Tabelle S1). Die Patient*innen wurden mit einer einmaligen Gabe von Reslizumab 1 mg oder 3 mg/kgKG i.v. behandelt. Die Effekte wurden bis zu 36 Wochen nach Injektion analysiert. Die eingeschlossenen Patient*innen waren zwischen 43 und 48 Jahre alt und zeigten Symptome trotz Standardtherapie (INS, oGKS oder Operationen der Nasennebenhöhlen). Neben CRScNP lag bei 18 von 24 Patient*innen ein Asthma und bei 2 Patient*innen eine Analgetikaintoleranz vor. Das Studiendesign war zur Klärung der Sicherheit und Pharmakokinetik ausgelegt. Daneben wurden folgende Messwerte erhoben: NPS, Nasal Symptom Score, NPIF, periphere Blutmarker sowie Biomarker im nasalen Sekret. Bei einem hohen Anteil der Patient*innen ließ sich eine Reduktion der Polypengröße bis 4 Wochen nach einmaliger Injektion detektieren. Die Responder konnten anhand der nasalen IL-5-Konzentration vorhergesagt werden. Derzeit besteht keine weitere Studienaktivität zur Zulassung dieses Antikörpers in dieser Indikation.

## 4. Einsatz der Biologika in der Routineversorgung

### Empfehlung #1

In dieser Indikation zugelassene Biologika sollen bei erwachsenen Patienten mit schwerer CRScNP bei fehlender Krankheitskontrolle als Zusatztherapie zu intranasalen Kortikosteroiden erwogen werden, wobei präparatespezifische Zulassungskriterien zu beachten sind (Konsens, Zustimmung 88 %).

**Figure 1:**
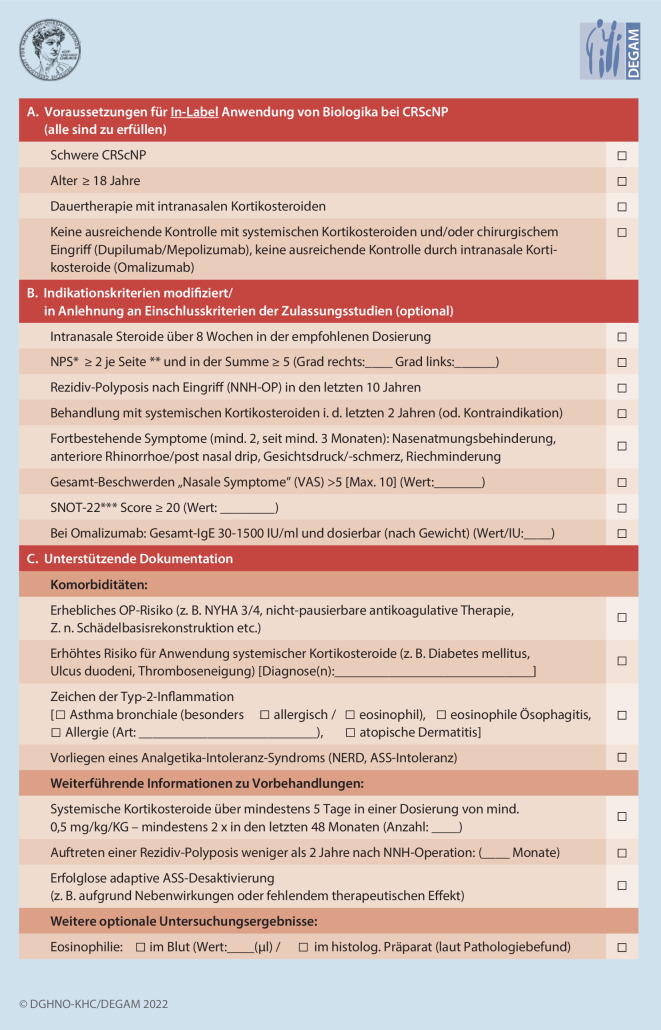

Alle 3 zur Zeit der Entwicklung dieses LL-Kapitels in Deutschland zugelassenen Biologika sind nur als Zusatztherapie bei gleichzeitiger Anwendung von INS zugelassen (Tab. 1; Tabelle S1; Empfehlung #1). Mögliche Kriterien und Instrumente für eine Erfassung der Krankheitskontrolle sind in Tab. 2 und in dem Dokumentationsbogen (Abb. 1 bzw. ESM 3) genannt. An diesen Minimalkriterien kann sich die Anwendung von Biologika orientieren, allerdings sollte in allen Fällen die individuelle Krankheitslast des/der Patient*in berücksichtigt werden. Aufgrund des Wirtschaftlichkeitsgebots für Verordnungen im deutschen GKV-System muss die Verordnung von Biologika ausreichend, zweckmäßig, und notwendig erscheinen.

**Table 1:** 

*Dupilumab*	„... ist angezeigt als Add-on-Therapie mit intranasalen Kortikosteroiden zur Behandlung von Erwachsenen mit schwerer CRSwNP, die mit systemischen Kortikosteroiden und/oder chirurgischem Eingriff nicht ausreichend kontrolliert werden kann.“ [8]
*Omalizumab*	„... als Zusatztherapie zu intranasalen Kortikosteroiden (INCS) zur Behandlung von Erwachsenen (ab 18 Jahren) mit schwerer CRSwNP angewendet, bei denen durch eine Therapie mit INCS keine ausreichende Krankheitskontrolle erzielt wird.“ [14]
*Mepolizumab*	„... angezeigt als Zusatztherapie mit intranasalen Kortikosteroiden zur Behandlung von erwachsenen Patienten mit schwerer CRSwNP, die mit systemischen Kortikosteroiden und/oder chirurgischem Eingriff nicht ausreichend kontrolliert werden kann.“ [21]

Die Fachinformation gibt für die bislang zugelassenen Präparate eine unterschiedliche Indikation für den Einsatz an – speziell im Hinblick auf eine nötige oder mögliche operative Vorbehandlung bzw. frühere Therapieversuche mit oGKS.

Dies bedeutet, dass zunächst über eine ausreichende Dauer die Standardtherapie durchzuführen ist und der jeweiligen Indikation Grenzen gesetzt sind.

Das Versagen der Standardtherapie (auch eine unzureichende Krankheitskontrolle [bzw. „schwere CRS“] genannt) wurde im Rahmen verschiedener europäischer LL und Positionspapiere [3, 4, 27] mit dem objektiven Nachweis von Polypen sowie einem starken subjektiven Leidensdruck gleichgesetzt. Zudem wurden in diesen Konsensuspapieren weitere Faktoren formuliert, die die Wahrscheinlichkeit eines Therapieansprechens steigern. Ein europäisches Expertenforum (*European Forum for Research and Education in Allergy and Airway Diseases [EUFOREA]*) hält in diesem Zusammenhang eine Behandlung mit Biologika angezeigt, wenn 4 der 6 in Tab. 2 aufgeführten Kriterien vorliegen [4].

**Table 2:** 

Rezidivpolypen nach NNH-OP
Vorliegen eines Asthma bronchiale
Nachweis einer Typ-2-Inflammation
Frustrane Vorbehandlung mit systemischen Steroiden (2 × im letzten Jahr)
Beeinträchtigung des Riechsinns
Beeinträchtigung der Lebensqualität

Dieses Vorgehen entspricht den Empfehlungen im *European Position Paper on Rhinosinusitis and Nasal Polyps *(*EPOS) *[3].

Bei der Beachtung dieser Vorgaben und der Anwendung in der klinischen Routine ergeben sich einige Unschärfen und Unterschiede. So ist beispielsweise eine vorherige Operation oder eine vorherige Behandlung mit oGKS für den Einsatz von Omalizumab nicht erforderlich, bei Dupilumab und Mepolizumab ist dies eine Voraussetzung. Auch sind die geforderten Schwellenwerte z. B. im SNOT-22 oder in den empfohlenen visuellen Analogskalen (VAS) nicht validiert, und deren Einsatz kann im Einzelfall eingeschränkt sein (kognitive Einschränkungen, fremdsprachliche Patient*innen, Lizenzfragen der angewendeten Fragebögen). Dies zeigt, dass die Kriterien weiterhin Gegenstand der wissenschaftlichen Diskussion sind und die zugrunde liegende Evidenz bislang begrenzt ist. Die sorgfältige Dokumentation des Schweregrads der CRScNP zur Indikationsstellung und Einleitung der Biologikatherapie ist daher von wesentlicher Bedeutung. Diese dient einerseits einer wirtschaftlichen Verordnung und andererseits der Beurteilung eines Therapieansprechens (Empfehlung #2). Hierbei kann der vorgeschlagene „*Dokumentationsbogen*“ (Abb. 1) hilfreich sein.

### Empfehlung #2

Der Schweregrad der Erkrankung sollte durch die Erhebung objektiver und subjektiver Kriterien vor Therapieeinleitung mit Biologika dokumentiert werden (starker Konsens, Zustimmung 100 %).

Überprüfung des Therapieerfolgs:

Es ist sinnvoll, eine Wirksamkeit bzw. die Fortführung des Einsatzes der Biologika nach einem angemessenen Zeitraum (z. B. spätestens nach einer Therapiedauer von einem halben Jahr nach Therapiebeginn [je nach Biologikum]) und im weiteren Verlauf regelmäßig zu überprüfen, um eine unnötige Dauerbehandlung bei Nonrespondern zu vermeiden (Empfehlung #3). Die klinisch relevante Wirksamkeit wird dabei subjektiv wie objektiv mit dem Befund vor Therapieeinleitung verglichen. Hierbei kommen verschiedene Instrumente zur Beurteilung der Krankheitskontrolle zum Einsatz (Tab. 3). Bei ausbleibendem Ansprechen ist ggf. ein Wechsel auf ein anderes Biologikum möglich.

**Table 3:** 

Lebensqualität
Subjektives Riechvermögen
Nasale Polypengröße
Komorbiditäten
Orale Steroidgabe
Riechtest
Rhinologische Funktionsdiagnostik
Lungenfunktionstest

### Empfehlung #3

Die Wirksamkeit einer Therapie mit Biologika bei CRScNP sollte nach einem angemessenen Zeitraum überprüft werden (Konsens, Zustimmung 88 %).

## 5. Kontraindikationen für den Einsatz von Biologika

Gegen den Einsatz von Biologika bei CRScNP sprechen diverse (relative) Kontraindikationen, wobei die Fach- und Gebrauchsinformationen der entsprechenden Präparate zu berücksichtigen sind (Empfehlung #4):

### Empfehlung #4

Bei Vorliegen von relativen Kontraindikationen sollte nur nach differenzierter Abwägung durch erfahrene Ärzt*innen und als Einzelfallentscheidung ein Therapieversuch mit Biologika eingeleitet werden (Konsens, Zustimmung 88 %).

## 6. Off-Label-Einsatz

Auch wenn im Einzelfall (beispielsweise bei speziellem Risikoprofil für einen operativen Eingriff wie eine fehlende Narkosefähigkeit, ein schlechter Allgemeinzustand, minderjährige Patienten o. Ä.) die Indikation nicht gemäß dem Zulassungstext gestellt werden kann, kann ein Therapieversuch mit den o. g. Biologika sinnvoll sein (Empfehlung #5). In diesem Zusammenhang ist auch die mittel- und langfristige Einsparung von Kosten in die Entscheidung für die Off-Label-Verordnung mit einzubeziehen. Diese erfordert allerdings eine umfängliche Dokumentation und Begründung vor Therapiebeginn gegenüber dem Kostenträger.

### Empfehlung #5

Auch wenn im Einzelfall die Indikation nicht gemäß dem Zulassungstext gestellt werden kann, kann ein individueller Therapieversuch mit den o. g. Biologika sinnvoll sein (Konsens, Zustimmung 88 %).

## 7. Dokumentationsbogen zur Therapie mit Biologika bei CRScNP

Wie oben ausgeführt, gibt es Kritikpunkte zu den von EUFOREA geforderten Minimalkriterien für den Einsatz von Biologika bei CRScNP. Vor diesem Hintergrund können ergänzend zu den o. g. allgemeinen, indikationsbedingten Voraussetzungen (Empfehlung #2) zur Demonstration der Schwere der Erkrankung bzw. zu fehlenden Alternativen in der Therapie diverse Faktoren herangezogen werden, um die Verordnung zu rechtfertigen. Um diese entsprechend zu erfassen, kann der in diesem LL-Kapitel vorgeschlagene Dokumentationsbogen (Abb. 1 bzw. ESM 3) zur Anwendung kommen, welcher eine Weiterentwicklung eines bereits bestehenden Dokumentationsbogens eines deutschsprachigen Positionspapiers darstellt (Empfehlung #6) [5].

### Empfehlung #6

Zur standardisierten Dokumentation von verschiedenen Aspekten zur Indikationsstellung und zur Verlaufskontrolle des Einsatzes von Biologika bei CRScNP sollte ein Dokumentationsbogen verwendet werden (Konsens, Zustimmung 88 %).

## Supplementary Information







## Abkürzungen

AWMF: Arbeitsgemeinschaft der wissenschaftlichen medizinischen Fachgesellschaften
CRScNP: Chronische Rhinosinusitis mit (cum) Nasenpolypen, deutscher Terminus
CRSwNP: Chronic Rhinosinusitis with Nasal Polyps, englischer Terminus
CT: Computertomographie
DEGAM: Deutsche Gesellschaft für Allgemeinmedizin und Familienmedizin
DGHNO-KHC: Deutsche Gesellschaft für Hals-Nasen-Ohren-Heilkunde, Kopf- und Hals-Chirurgie
EPOS: European Position Paper on Rhinosinusitis and Nasal Polyps
EUFOREA: European Forum for Research and Education in Allergy and Airway Diseases
GRADE: Grading of Recommendations, Assessment, Development and Evaluations
HrQoL: Health-related Quality of Life/gesundheitsbezogene Lebensqualität
IgE: Immunglobulin E
IL: Interleukin
INS: Intranasale Steroide
IQWiG: Institut für Qualität und Wirtschaftlichkeit im Gesundheitswesen
LL: Leitlinie
LMK: Lund-Mackay-Score
NCS: Nasal Congestion Score/Nasaler Obstruktions-Score
NPS: Nasal Polyp Score/Nasaler Polypen-Score
oGKS: Orale Glukokortikosteroide
PNIF: Peak Nasal Inspiratory Flow
SNOT: Sino-Nasal Outcome Test
s.c.: Subkutan
UPSIT: University of Pennsylvania Smell Identification Test
VAS: Visuelle Analogskala
zLMK: Nach Zinreich modifizierter LMK
