# Supplementary material for: Treatment of chronic rhinosinusitis with nasal polyps (CRSwNP) with monoclonal antibodies (biologics): S2k guideline of the German Society of Oto-Rhino-Laryngology, Head and Neck Surgery (DGHNO-KHC), and the German College of General Practitioners and Family Physicians (DEGAM)
Source: HNO. 2023 Mar 20;71(4):256–63. [Article in German] doi: 10.1007/s00106-023-01273-2 (PMC10066152; doi:10.1007/s00106-023-01273-2)
Supplement: Supplementary file 1 [file 106_2023_1273_MOESM1_ESM.pdf]

ESM 1- systematische Aufbereitung der Evidenz

Tabelle 1-3: Recherche, Auswahl und kritische Bewertung

zu

Therapie der chronischen Rhinosinusitis mit Polyposis nasi (CRScNP) mit monoklonalen Antikörpern (Biologika): S2k-Leitlinie der Deutschen Gesellschaft für Hals-Nasen-Ohren-Heilkunde, Kopf- und Hals-Chirurgie (DGHNO-KHC) und der Deutschen Gesellschaft für Allgemeinmedizin und Familienmedizin (DEGAM).

Tabelle 1

Evidenztabelle der Aktualisierung des Kapitels „Therapie der CRScNP mit Biologika"

Autor, Jahr, Studien-Code	Studien-Design (Anzahl Patienten)	Alter (Jahre) (Intervention vs. Vergleichsgruppe)	Population und Vorerkrankungen	Intervention	Komparator	Follow up	Effektstärke (=primäre und sekundäre Endpunkte)	Evidenzgrad (Oxford-Kriterien 2009*)
**Dupilumab**								
Bachert et al. Lancet. 2019; 394(10209): 1638-1650 LIBERTY-SINUS 24	Multizentrische RCT (N=276, 2-armig)	Mittelwert 50 (41-60) (Placebo) vs. 52 (39-61)	bilaterale CRSwNP und Symptome trotz INC und oraler GKS-Gabe in den letzten 2 Jahren oder FEES, 57% Asthmapatienten in der Verumgruppe	Dupilumab subkutan 300 mg (alle 2 Wo.), 24 Wochen	Placebo	24 Wochen	LS Mittelwertunterschiede zwischen den beiden Gruppen<br><br>NPS: -2,06; p<0,0001<br><br>NCS: -0,89; p<0,0001<br><br>LMK –7,44; p<0,0001<br><br>TSS –2,61; p<0,0001<br><br>UPSIT: 10,56; p<0,0001<br><br>LOS: –1,12; p<0,0001<br><br>SNOT-22: –21,12; p<0,0001	1b

Bachert et al.Lancet. 2019; 394(10209): 1638-1650 LIBERTY-SINUS 52	Multizentrische RCT (N=448, 3-armig)	Mittelwert 53 (44-61) (Placebo) vs. 53 (42-63) (Gruppe 2) vs. 51 (42-61) (Gruppe 3)	bilaterale CRSwNP und Symptome trotz INC und oraler GKS-Gabe in den letzten 2 Jahren oder endonasaler NNH-Operation (FEES), 60 % Asthmapatienten in der Verumgruppe	Gruppe 2: Dupilumab subkutan 300 mg (alle 2 Wo.), 52 Wochen<br>Gruppe 3: Dupilumab 300 mg (alle 2 Wo.), 24 Wo., dann 300 mg (alle 4 Wo.) über 42 Wochen	Placebo (Gruppe 1)	52 Wochen	LS Mittelwertunterschiede zwischen Gruppe 2 und Placebo in Woche 24<br><br>NPS: -1,80; p < 0,0001<br><br>NCS: -0,87;p < 0,0001<br><br>LMK –5,13; p < 0,0001<br><br>TSS –2,44; p < 0,0001<br><br>UPSIT: 10,52; p < 0,0001<br><br>LOS: –0,98; p < 0,0001<br><br>SNOT-22: –17,36; p < 0,0001<br><br>LS Mittelwertunterschiede zwischen Gruppe 2 und Placebo in Woche 52<br><br>NPS: -2,40; p<0,0001<br><br>NCS: -0,98; p<0,0001<br><br>SNOT-22: -20,96; p<0,0001	1b
Bachert et al.: Effect of Subcutaneous Dupilumab on Nasal Polyp Burden in Patients With Chronic Sinusitis and Nasal Polyposis A Randomized Clinical Trial. JAMA. 2016;315(5):469-479. doi:10.1001/jama.2015.19330	Multizentrische RCT (N=276, 2-armig)	Mittelwert (SD) 49.3 (9,1) (Placebo) vs. 47,4 (9,8)	18 jährige (bis 65jährige) Patienten mit bilateraler CRSwNP und chronischen Symptomen trotz intranasalem Steroid (INC) für mindestens 2 Monate, 53% Asthmapatienten in der Verumgruppe	Dupilumab subkutan 600 mg als Startdosis, dann 300 mg (wöchentlich Wo.), insgesamt 16 Wochen	Placebo	16 Wochen	LS Mittelwertunterschiede zwischen den beiden Gruppen<br><br>NPS: -1,6; p<0,001<br><br>LM-CT Score: -8.8; p<0,001<br><br>SNOT-22: -18.1; p<0,001<br><br>UPSIT: 14.8; p<0,001	1b

Bachert et al. Allergy. 2020;75: 148–157	Multizentrische RCT (N=60, 2-armig)<br><br>VAS und Lebensqualität (Sekundäre Endpunkte)	Mittelwert (SD) 49.3 (9,1) (Placebo) vs. 47,4 (9,8)	18 jährige (bis 65jährige) Patienten mit bilateraler CRSwNP und chronischen Symptomen trotz intranasalem Steroid (INC) für mindestens 2 Monate, 53% Asthmapatienten in der Verumgruppe	Dupilumab subkutan 600 mg als Startdosis, dann 300 mg (wöchentlich Wo.), insgesamt 16 Wochen	Placebo	16 Wochen	Sekundäre Endpunkte aus Bachert et al. Allergy. 2020;75:148–157<br><br>VAS > 3-10 Verum: 82.2% (Baseline) zu 21.4%<br>Placebo: 88.0% (Placebo) zu 84.2%<br><br>SF-36 in 7 Domänen Verbesserung zu Baseline; Placebogruppe in 3 Domänen<br><br>Weniger Krankheitstags (0,09) als in Placebogruppe (4.18; p=0,015)	1b
Bachert et al. Rhinology 2020; 58: 1, 1 -17	Multizentrische RCT (N=60, 2-armig)<br><br>CT-Scores	Mittelwert (SD) 49.3 (9,1) (Placebo) vs. 47,4 (9,8)	18 jährige (bis 65jährige) Patienten mit bilateraler CRSwNP und chronischen Symptomen trotz intranasalem Steroid (INC) für mindestens 2 Monate, 53% Asthmapatienten in der Verumgruppe	Dupilumab subkutan 600 mg als Startdosis, dann 300 mg (wöchentlich Wo.), insgesamt 16 Wochen	Placebo	16 Wochen	Sekundäre Endpunkte aus Bachert et al. Allergy. 2020;75:148–157<br><br>LS Mittelwertunterschiede zwischen den beiden Gruppen für den Standard LMK -8.8; p<0,0001 zLMK-score -15.4; p<0,0001	1b

© DGHNO-KHC/DEGAM 2022

Omalizumab								
Gevaert et al. J Allergy Clin Immunol 2020;146:595-605. NCT03280550 POLYP 1	Multizentrische RCT (N=138, 2-armig)	Mittelwert (SD) 52.2 (11.6) (Placebo) vs. 50.0 (14.5)	18 jährige (bis 75 jährige) Patienten mit bilateraler CRSwNP und Symptomen trotz INC und einem Körpergewicht (KG) und IgE-Spiegel, der den Einsatz von Omalizumab (Fach-und Gebrauchsinformation (FG)) erlaubt.	Omalizumab 75 mg bis 600 mg, subkutan alle 2 oder 4 Wochen (nach KG und IgE).	Placebo	24 Wochen	Mittelwertunterschiede zwischen den beiden Gruppen im Vergleich zum Basiswert:<br><br>NPS: -1.14; p<0,001<br><br>NCS: -0,55; p=0,0004<br><br>SNOT-22: -16,12; p<0,0001<br><br>UPSIT: 3,81; p=0,0024<br><br>TNSS: -1,91; p=0,0001<br><br>LOS: -0,33; p=0,161<br><br>Postnasal Drip Score: -0,56; p=0,0001<br><br>Runny Nose Score: -0,43; p=0023	1b

© DGHNO-KHC/DEGAM 2022

Gevaert et al. JAllergy Clin Immunol 2020; 146:595-605. NCT03280550 POLYP 2	Multizentrische RCT (N=127, 2-armig)	Mittelwert (SD) 51.0 (12) (Placebo) vs. 49 (11,9)	18 jährige (bis 75 jährige) Patienten mit bilateraler CRSwNP und Sympto-men trotz INC und einem Körpergewicht (KG) und IgE-Spiegel, der den Einsatz von Omalizumab (Fach-und Gebrauchsinformation (FG)) erlaubt.	Omalizumab 75 mg bis 600 mg, subkutan alle 2 oder 4 Wochen (nach KG und IgE).	Placebo	24 Wochen	Mittelwertunterschiede zwischen den beiden Gruppen im Vergleich zum Basiswert:<br><br>NPS: -0,59; p=0,014<br><br>NCS: -0,50; p=0,0017<br><br>SNOT-22: -15,04; p <0,0001<br><br>UPSIT: 3,86; p=0,0011<br><br>TNSS: -2,09; p<0,0001<br><br>LOS: -0,45; p=0024<br><br>Postnasal Drip Score: -0,54; p=0,0001<br><br>Runny Nose Score: -0,63; p<0,0001	1b
Chandra et al.: Int Forum Allergy Rhinol. 2016;6:472–477	retrospektive Auswertung (N=25)	Mittelwert (SD) 47,2 (16,4)	25 Asthmatiker, hiervon 8 Patienten mit Polyposis	Omalizumab 75 mg bis 600 mg, subkutan alle 2 oder 4 Wochen (nach KG und IgE).	kein	4-12 Wochen	Antibiotika-Verordnung pro Monat um 37 % ggü. Vortherapie reduziert (p=0,013)<br><br>Gabe von systemischen GKS pro Monat in 8 von 19 Patienten reduziert	4/5 (retrospek-tive Aus-wertung von Patienten-akten)

Bidder et al. Omalizumab treats chronic rhinosinusitis with nasal polyps and asthma together-a real life study Rhinology. 2018 Mar 1;56(1):42-45. doi: 10.4193/Rhin17.139	Prospektiv, deskriptiv (N=37)	Mittelwert 46 (Omalizumab) vs. 48 (FEES)	CRSwNP (definiert als SNOT-22>50) und schweres allergisches Asthma	Gruppe 1:<br>13 Patienten: Omalizumab<br>75 mg bis 600 mg, subkutan alle 2 oder 4 Wochen (nach KG und IgE).<br><br>Gruppe 2:<br>24 Patienten: FEES	Placebo	16 Wochen	Gegenüber Baseline in Woche 16:<br>TPS: -2.67 Omalizumab (p=0,001), -0,12 Placebo (nicht signifikant)<br><br>LMK -4 Omalizumab (p=0,02), +0,5 Placebo (nicht signifikant)<br><br>nasale Symptome und Dyspnoe signifikant besser in Omalizumab (P 0,02)<br><br>SF-36 signifikant besser in Omalizumab (p=0,02)<br><br>RSOM-31 Schlaf und generelle Symptome signifikant besser in Omalizumab (p=0,01), keine Abhängigkeit der Ergebnisse von Analgetikaintoleranz	1b

© DGHNO-KHC/DEGAM 2022

Gevaert et al. Omalizumab is effective in allergic and nonallergic patients with nasal polyps and asthma. J Allergy Clin Immunol. 2013 Jan;131(1):110-6.e1	Bizentrisch RCT (N=24, 2-armig)	Median 45 (42-54) (Placebo) vs. 50 (44-56)	18 jährige (und ältere) Patienten mit CRSwNP und Asthma, 12/24 Patienten mit Analgetika Intoleranz Syndrom	Omalizumab 75 mg bis 375 mg, subkutan alle 2 oder 4 Wochen (nach KG und IgE).	Placebo	16 Wochen	Gegenüber Baseline in Woche 16: TPS: -2.67 Omalizumab (p=0,001), -0,12 Placebo (nicht signifikant)<br><br>LMK -4 Omalizumab (p=0,02), +0,5 Placebo (nicht signifikant)<br><br>nasale Symptome und Dyspnoe signifikant besser in Omalizumab (P 0,02)<br><br>SF-36 signifikant besser in Omalizumab (p=0,02)<br><br>RSOM-31 Schlaf und generelle Symptome signifikant besser in Omalizumab (p=0,01), keine Abhängigkeit der Ergebnisse von Analgetikaintoleranz	1b
Pinto et al. A randomized, double-blind, placebo-controlled trial of anti-IgE for chronic rhinosinusitis. Rhinology . 2010 Sep;48(3):318-24	Monozentrisch randomisiert kontrolliert (RCT) (N=14, 2-armig)	Mittelwert (SD) 49 (9) (Placebo) vs. 43 (10)	18 jährige (bis 65jährige) Patienten mit CRS	Omalizumab 150 mg bis 375 mg, subkutan alle 2 oder 4 Wochen (nach KG und IgE)	Placebo	6 Monate	Gegenüber Baseline: Verbesserung in NNH-CT: Verschattungen der Sinus (p>0,043) in Omalizumab, Placebo n.s.	1b
Penn et al. The role of anti-IgE immunoglobulin therapy in nasal polyposis: a pilot study. Am J Rhinol . Jul-Aug 2007;21(4):428-32	Monozentrisch retrospektiv, (N=8, 2-armig)	Median 28 (Placebo) vs. 40)	18 jährige (und ältere) Patienten mit NP und atopischem Asthma und allergischer Rhinosinusitis	Omalizumab 150 mg bis 375 mg, subkutan alle 2 oder 4 Wochen (nach KG und IgE)	Post-OP SOC	9 Monate (Omalizumab) bzw. 10,5 Monate (SOC)	Gegenüber Baseline:<br><br>NPS Besserung auf 0,25 (von 2,00) signifikant (p=0,03), SOC n.s	4

© DGHNO-KHC/DEGAM 2022

**Mepolizumab**								
Han et al. Mepolizumab for chronic rhinosinusitis with nasal polyps: a randomised, double-blind, placebo-controlled, phase 3 trial. Lancet Repir Med. 2021 Oct;9(10):1141-1153. doi: 10.1016/ S2213- 2600(21) 00097-7 (SYNAPSE	multizentrisch RCT (N=407, 2-armig)	Mittelwert (SD) 49 (13) (Placebo) vs. 49 (14)	18 jährige (und ältere) Patienten mit rezidivierender therapierefraktärer, symptomatische, schwerer beidseitige CRSwNP; Asthmapatienten Placebo 149 (74 %), Verum 140 (68 %) Ass-Intoleranz: Placebo 63 (31%), Verum 45 (22 %)	Mepolizumab 100 mg s.c. alle 4 Wochen als Zusatztherapie zur Standardtherapie	Placebo	49-52 Wochen	LS Mittelwertunterschiede zwischen den beiden Gruppen in Woche 49-52<br><br>NPS: -0,73; p<0,0001<br><br>VAS Nasale Obstruktion: -3,41; p<0,0001<br><br>Notwendigkeit einer FEES: Mepolizumab 9 %, Placebo 23 %; p< 0,0032<br><br>VAS Symptomscore: -3,18; p< 0,0032<br><br>SNOT-22: -16.49; p=0,0032<br><br>Anteil an Patienten mit oraler GKS: Mepolizumab 25 %, Placebo 37 %; p=0,020<br><br>Kombinierter VAS: -2,68; p=0,02<br><br>VAS LOS: -0,37; p=0,02	1b

© DGHNO-KHC/DEGAM 2022

Kurosawa et al. Favorable clinical efficacy of mepolizumab on the upper and lower airways in severe eosinophilic asthma: a 48-week pilot study. Eur Ann Allergy Clin Immunol. 2019 Sep 16;51(5):213-221	Monozentrische, open-label Fallserie (n=11)	Jahre (Median): Männer: 55 (29-69), Frauen 50 (44-56)	schweres eosinophiles Asthma und CRS, Analgetika-intoleranz bei 6 Patienten	Mepolizumab 100 mg, subkutan alle 4 Wochen	n/v	48 Wochen	Gegenüber Baseline:<br><br>SNOT-22: -18; p<0,01<br><br>SNOT-Item Anosmie: -2,5; p<0,05<br><br>SNOT-item: Nasale Verstopfung: -1,9; p<0,01<br><br>LMK: -3.0; p>0,01<br><br>FEV1: +4,3% (Wo.24) und +4.9% (Wo.48)	4
Bachert et al. Reduced need for surgery in severe nasal polyposis with mepolizumab: Randomized trial. J Allergy Clin Immunol. 2017 Oct;140(4):1024-1031	multizentrisch RCT (N=109, 2-armig)	Median 50 (Placebo) vs. 51	18 jährige (bis 70 jährige) Patienten mit bilateraler schwerer eosinophiler NP trotz INC und Indikation zur FEES, Asthma 81 % (Verum) vs. 75 % (Placebo)	Mepolizumab 750 mg, intravenös alle 4 Wochen	Placebo	25 Wochen	In Woche 25 keine Indikation zur FEES: Mepo n=16 (30 %) vs Placebo n=5 (10 %); p=0,006; signifikant ab Woche 9<br><br>Mittelwertunterschiede zwischen den Gruppen in Woche 25<br><br>VAS-Rhinorrhoe: -2,3; p<0,001<br><br>VAS-Mucus: -2,1; p<0,001<br><br>VAS-Nasale Obstruktion: -1,8; p=0,002<br><br>VAS LOS: -1,9; p<0,001<br><br>SNOT-22: -13.2; p= 0,005	1b

© DGHNO-KHC/DEGAM 2022

Gevaert et al.Mepolizumab, a humanized anti-IL-5 mAb, as a treatment option for severe nasal polyposis.J Allergy Clin Immunol . 2011 Nov;128(5):989-95	Monozentrisch RCT (N=30, 2-armig)	Mittelwert (SD) 46 (11) (Placebo) vs. 50 (9)	18 jährige (und älter) Patienten mit schwerer, therapierefraktär NP, kein SOC-Behandlung, Asthma 50 % in Verum-, 30 % in Placebogruppe	Mepolizumab 750 mg, intravenös 2 mal im Abstand von 4 Wochen	Placebo	8 Wochen	Mittelwertunterschiede in Woche 8<br><br>NPS -1,30; p=0,028<br><br>NNH-CT: Verbesserung mehr als 50% in Mepo vs. 20% Placebo; p=0,049	1b
**Reslizumab**								
Gevaert et al. Nasal IL-5 levels determine the response to anti–IL-5 treatment in patients with nasal polyps. JAllergy Clin Immunol. 2006 Nov;118(5):1133-41	Bizentrisch RCT (N=24, 3-armig)	Median 48 (21-59) (Placebo) vs. 44 (22-63) (Gruppe 1) vs. 49 (18-57) (Gruppe 3)	18 jährige (und älter) Patienten mit schwerer, therapierefraktär NP Asthmapatienten 75 %, kein SOC-Behandlung,	Gruppe 1: Einmalige Gabe Reslizumab (1mg/kgKG) intravnös oder Gruppe 2: Einmalige Gabe Reslizumab (3mg/kgKG) intravenös	Placebo	36 Wochen	Klinische Parameter: Keine signifikanten Unterschiede zwischen den drei Gruppen	1b

* nach Bob Phillips, Chris Ball, Dave Sackett, Doug Badenoch, Sharon Straus, Brian Haynes, Martin Dawes (November 1998). Aktualisiert von Jeremy Howick im März 2009

Abkürzungen: CRS, chronische Rhinosinusitis; CRScNP, chronische Rhinosinusitis mit Polypen (chronische Rhinosinusitis with Polyps); CT, Computertomographie; FEES: funktionelle endoskopische Nasennebenhöhlen-Operation (functional endoscopic sinus surgery); FEV-1, Funktionelle Einsekundenkapazität; GKS, Glukokortikosteroid; HQoL, Krankheitsbezogenen Lebensqualität (Health Related Quality of Life); INC: intranasales Kortikosteroid (intranasal Corticosteroid); IU, Internationale Einheiten (International Units); KG: Körpergewicht; LMS: Lund-Mackay-Score; NCS: Nasaler Obstruktions Score (Nasal Congestion Score); LOS, Riechminderung (Loss of Smell); NPIF, Nasale Inspiratorischer Spitzenfluss (Nasal Peak Inspiratory Flow); NP, Nasale Polyposis (Nasal Polyps); RCT: randomisierte, kontrollierte Studien (randomized controlled trials); RSOM-31: Rhinosinusitis Outcome Measuring Instrument-31; SD: Standardabweichung (Standard Deviation); SF-63, Short Form 36; SNOT-20: Sino-Nasal Outcome Test-20; SOC, Standardbehandlung (Standard of Care); TSS, Total Symptom Score; TNSS, Total Nasal Symptom Score; TPS, Total (Nasal Endoscopic) Polyp Score; zLMK: Zinreich-Mackay score; UPSIT, University of Pennsylvania Smell Identification Test

© DGHNO-KHC/DEGAM 2022

Tabelle S2: Terms für systematische Recherche

Ovid MEDLINE(R) and Epub Ahead of Print, In-Process & Other Non-Indexed Citations, Daily and Versions(R) 1946 to August 20, 2020

1 exp Sinusitis/
2 paranasal sinus diseases/ or rhinitis/ or rhinitis, atrophic/ or rhinitis, vasomotor/
3 exp Paranasal Sinuses/
4 (rhinosinusitis or nasosinusitis or pansinusitis or ethmoiditis or sphenoiditis).ab,ti.
5 (kartagener* adj3 syndrome*).ab,ti.
6 (inflamm* adj5 sinus*).ab,ti.
7 ((maxilla* or frontal*) adj3 sinus*).ab,ti.
8 1 or 2 or 3 or 4 or 5 or 6 or 7
9 exp chronic disease/
10 exp Recurrence/
11 (chronic or persis* or recur*).ab,ti.
12 9 or 10 or 11
13 8 and 12
14 CRSsNP.ab,ti.
15 ((sinusitis or rhinitis) adj3 (chronic or persis* or recur*)).ab,ti.
16 13 or 14 or 15
17 exp Nasal Polyps/
18 exp Nose/ or exp Nose Diseases/
19 exp Polyps/
20 18 and 19
21 ((nose or nasal or rhino* or rhinitis or sinus* or sinonasal) adj3 (papilloma* or polyp*)).ab,ti.
22 (rhinopolyp* or CRSwNP).ab,ti.
23 16 or 17 or 20 or 21 or 22
24 exp Antibodies, Monoclonal/
25 exp Antibodies, Anti-Idiotypic/
26 exp Immunoglobulin E/
27 exp INTERLEUKINS/
28 exp Receptors, Interleukin/
29 exp Biological Therapy/
30 exp Granulocyte-Macrophage Colony-Stimulating Factor/
31 exp Cytokines/
32 exp Etanercept/ or exp Alefacept/
33 (Antibod* adj3 monoclonal).ab,ti.
34 (Interleukin* or IgE or "immunoglobulin E" or Antiglobulin* or antiidiotyp*).ab,ti.
35 (anti adj3 (globulin* or idiotyp* or immunoglobulin* or M1 or CCR4 or "LFA 1" or "GATA 3" or OX40L)).ab,ti.
36 (ralokimumab or Adalimumab or Alemtuzumab or Bevacizumab or Certolizumab or Cetuximab or Denosumab or Ipilimumab or Natalizumab or Omalizumab or Palivizumab or Ranibizumab or Trastuzumab or stekinumab or mepolizumab or Nucala or SB240563 or "SB 240563" or dupilumab or REGN668 or AMG317 or "AMG 317" or AMG827 or "AMG 827" or DNAzyme or antiTSLP or CSL311 or "CSL 311" or "AMG 761" or AMG761 or "AMG 837" or KW0761 or "KW 0761" or "CSF 2" or "CSF GM").ab,ti.
37 (siliq or D2E7 or humira or campath or Lemtrada or avastin or cimzia or CDP870 or "CDP 870" or Erbitux or C225 or Xgeva or prolia or "AMG 162" or AMG162 or Yervoy or Tysabri or Antegren or Xolair or Synagis or Rhu-Fab or lucentis or Herceptin or stelara or

CNTO or ASM8 or granulocyte-macrophage or GM-CSF or QGE031 or Raptiva or AK001 or "AK001").ab,ti.
38 ((B-cell or T-cell or Eosinophil or "mast cell" or stimulating) adj3 factor).ab,ti.
39 (CD23 or CD2 or CD11a or CD20 or CD25 opr CD252 or (receptor* adj3 apsilon)).ab,ti.
40 (CD adj3 ("23" or antigen* or "2" or 11a or "20" or "25" or "252")).ab,ti.
41 ((antigamma or "anti gamma") adj3 Antibod*).ab,ti.
42 (IgEid or "55700" or SCH55700 or CEP38072 or "CEP 38072" or cinqair or DCP835 or "DCP 835" or GM-CSF or TNF or TSLP or OX40L).ab,ti.
43 (IL adj3 ("5" or five or "4" or four or "13" or thirteen or "1" or one or "10" or ten or "11" or eleven or "12" or twelve or "15" or fifteen or "16" or sixteen or "17" or seventeen or "18" or eighteen or "2" or two or "23" or "twenty three" or "12" or twelve or "27" or "twenty seven" or "3" or three or "33" or "thirty three" or "6" or six or "7" or seven or "8" or eight or "9" or nine or 5R* or 1R1 or 4R* or 12p40 or IL-23p40 or 17A or 17RA or "22" or "twenty two" or "31" or "thirty one" or 31R)).ab,ti.
44 (IL5 or IL4 or IL13 or IL1 or IL10 or IL11 or IL12 or IL15 or IL15 or IL16 or IL17 or IL18 or IL2 or IL23 or IL12 or IL27 or IL3 or IL33 or IL6 or IL7 or IL8 or IL9 or IL22 or IL31).ab,ti.
45 (biologic or biologics or biotherap*).ab,ti.
46 (biologic* adj3 therap*).ab,ti.
47 (mAB or mepo or MDX or MEDI or siglec* or "lectin 8").ab,ti.
48 (SAR231893 or reslizumab or siglec8 or benralizumab or lebrikizumab or brodalumab or Tralokinumab or Quilizumab or Ligelizumab or Mogamulizumab or Efalizumab or Pitrakinra or Odulimomab or Mogamulizumabor or BCGF or binetrakin or "anti antibod*").ab,ti.
49 (siglec8 or TPI ASM8 or Rilonacept).rn.
50 (Canakinumab or Ilaris or Rilonacept or Arcalyst or Anakinra or Kineret or Antril or Altrakincept or Nuvance or Pascolizumab or SB 240683 or VAK694 or QBX258 or VAK 694 or VAK-694 or dectrekumab QAX-576 or QAX576 or QAX 576 or aerovant or AER-001 or AER001 or "AER 001" or BAY-16-9996 or BAY 16-9996 or Bosatria or Nucala or CDP 835 or CDP835 or CDP-835 or CINQAIR or CTx55700 or CTx 55700 or CTx-55700 or DCP 835 or DCP-835 or DCP835 or SCH5570 or SCH 5570 SCH-5570 or TRFK-5 or TRFK 5 or TRFK5 or BIW-8405* or BIW8405* or BIW 8405* or KHK 4563 or KHK-4563 or KHK4563 or Enokizumab or 7F3com-2H2 or Ustekinumab or Stelara or CNTO-1275 or CNTO1275 or CNTO 1275 or Anrukinzuma* or IMA-638 or PF-05230917 or GSK679586 or GSK-679586 or GDK 679586 or IMA026 or IMA-026 or "IMA 026" or IMA638 or IMA 638 MILR1444A or MILR 1444A or MILR-1444A or PRO-301444 or PRO301444 or PRO 301444 or RG-3637 or RG3637 or RG 3637 or RO-5490255 or RO5490255 or RO5490255 or TNX-650 or TNX650 or TNX 650 or RPC-4046 or ABT-308 or RPC4046 or ABT308 or RPC 4046 or ABT 308 or CAT-354 or CAT354 or CAT 354 or Secukinumab or Cosentyx or AIN-457 or KB-03303A or NVP-AIN 457 or AIN457 or KB03303A or NVPAIN457 or AIN 457 or KB03303A or NVP-AIN-457 or KHK-4827 or KHK4827 or KHK 4827 or fezakinumab* or ILV-094 or PF-5212367 or ILV094 or PF5212367 or "ILV094" or PF 5212367 or BMS-981164 or BMS981164 or BMS 981164 or Nemolizumab or CIM331 or CIM 331 or CIM-331 or Lenzilumab or KB003 or "KB 003" or KB-003 or ABT-D2E7 or D2E7 or LU 200134 or ABTD2E7 or LU200134 or ABT D2E7 or LU 200134 or Golimumab or Simponi or CNTO-148 or CNTO148 or CNTO 148 or Inflixima or cA2 or CenTNF or Remicade or TA-650 or TA650 or TA 650 or Etanercept or Enbrel or p75TNFR-Ig or rhu TNFR-Fc or TNFR-Fc-p75 or TNR-001 or TNR001 or "TNR 001" or AMG-157 or MEDI-9929 or AMG157 or AMG 157 MEDI4212 or MEMP1972A or RG7449 or MEMP 1972A or RG 7449 or MEMP-1972A or RG-7449 or Mogamulizumab or KM8761 or Poteligeo or KM-

8761 or KM 8761 or Alefacept or Amevive or "ASP 0485" or BG 9273 or BG 9712 or ASP0485 or BG9273 or BG9712 or ASP-0485 or BG-9273 or BG-9712 or Xanelim or Rituximab or Rituxan or Daclizumab or Zenapax or Oxeluma* or huMAb or OX40L or RG4930 or RO4989991 or RG4930 or RG-4930 or RO4989991 or RO-4989991 or Bertilimumab or Tezepeluma or Isunakinra or "Fusion Protein*" or cytokine*).ab,ti.

51	or/24-50
52	23 and 51
53	randomized controlled trial.pt.
54	controlled clinical trial.pt.
55	randomized.ab.
56	placebo.ab.
57	drug therapy.fs.
58	randomly.ab.
59	trial.ab.
60	groups.ab.
61	53 or 54 or 55 or 56 or 57 or 58 or 59 or 60
62	exp animals/ not humans.sh.
63	61 not 62
64	52 and 63
65	limit 64 to (yr="2005 -Current" and (english or german))
66	exp adult/
67	adult*.ab,ti.
68	66 or 67
69	65 and 68

Tabelle S3: Gradeinteilung der Evidenz (nach Bob Phillips, Chris Ball, Dave Sackett, Doug Badenoch, Sharon Straus, Brian Haynes, Martin Dawes (November 1998). Aktualisiert von Jeremy Howick im März 2009.)

Level	Therapy / Prevention, Aetiology / Harm	Prognosis	Diagnosis	Differential diagnosis / symptom prevalence study	Economic and decision analyses
1a	SR (with homogeneity*) of RCTs	SR (with homogeneity*) of inception cohort studies; CDR" validated in different populations	SR (with homogeneity*) of Level 1 diagnostic studies; CDR" with 1b studies from different clinical centres	SR (with homogeneity*) of prospective cohort studies	SR (with homogeneity*) of Level 1 economic studies
1b	Individual RCT (with narrow Confidence Interval"¡)	Individual inception cohort study with > 80% follow-up; CDR" validated in a single population	Validating** cohort study with good" " " reference standards; or CDR" tested within one clinical centre	Prospective cohort study with good follow-up****	Analysis based on clinically sensible costs or alternatives; systematic review(s) of the evidence; and including multi-way sensitivity analyses
1c	All or none§	All or none case-series	Absolute SpPins and SnNouts" "	All or none case-series	Absolute better-value or worse-value analyses " " " "
2a	SR (with homogeneity*) of cohort studies	SR (with homogeneity*) of either retrospective cohort studies or untreated control groups in RCTs	SR (with homogeneity*) of Level >2 diagnostic studies	SR (with homogeneity*) of 2b and better studies	SR (with homogeneity*) of Level >2 economic studies
2b	Individual cohort study (including low quality RCT; e.g., <80% follow-up)	Retrospective cohort study or follow-up of untreated control patients in an RCT; Derivation of CDR" or validated on split-sample§§§ only	Exploratory** cohort study with good" " " reference standards; CDR" after derivation, or validated only on split-sample§§§ or databases	Retrospective cohort study, or poor follow-up	Analysis based on clinically sensible costs or alternatives; limited review(s) of the evidence, or single studies; and including multi-way sensitivity analyses
2c	"Outcomes" Research; Ecological studies	"Outcomes" Research		Ecological studies	Audit or outcomes research
3a	SR (with homogeneity*) of case-control studies		SR (with homogeneity*) of 3b and better studies	SR (with homogeneity*) of 3b and better studies	SR (with homogeneity*) of 3b and better studies

3b	Individual Case-Control Study		Non-consecutive study; or without consistently applied reference standards	Non-consecutive cohort study, or very limited population	Analysis based on limited alternatives or costs, poor quality estimates of data, but including sensitivity analyses incorporating clinically sensible variations.
4	Case-series (and poor quality cohort and case-control studies§§)	Case-series (and poor quality prognostic cohort studies***)	Case-control study, poor or non-independent reference standard	Case-series or superseded reference standards	Analysis with no sensitivity analysis
5	Expert opinion without explicit critical appraisal, or based on physiology, bench research or "first principles"	Expert opinion without explicit critical appraisal, or based on physiology, bench research or "first principles"	Expert opinion without explicit critical appraisal, or based on physiology, bench research or "first principles"	Expert opinion without explicit critical appraisal, or based on physiology, bench research or "first principles"	Expert opinion without explicit critical appraisal, or based on economic theory or "first principles"

Notes

Users can add a minus-sign "-" to denote the level of that fails to provide a conclusive answer because:

- EITHER a single result with a wide Confidence Interval
- OR a Systematic Review with troublesome heterogeneity.

Such evidence is inconclusive, and therefore can only generate Grade D recommendations.

* By homogeneity we mean a systematic review that is free of worrisome variations (heterogeneity) in the directions and degrees of results between individual studies. Not all systematic reviews with statistically significant heterogeneity need be worrisome, and not all worrisome heterogeneity need be statistically significant. As noted above, studies displaying worrisome heterogeneity should be tagged with a "-" at the end of their designated level.

" Clinical Decision Rule. (These are algorithms or scoring systems that lead to a prognostic estimation or a diagnostic category.)

"¡ See note above for advice on how to understand, rate and use trials or other studies with wide confidence intervals.

§ Met when all patients died before the Rx became available, but some now survive on it; or when some patients died before the Rx became available, but none now die on it.

§§ By poor quality cohort study we mean one that failed to clearly define comparison groups and/or failed to measure exposures and outcomes in the same (preferably blinded), objective way in both exposed and non-exposed individuals and/or failed to identify or appropriately control known confounders and/or failed to carry out a sufficiently long and complete follow-up of patients. By poor quality case-control study we mean one that failed to clearly define comparison groups and/or failed to measure exposures and outcomes in the same (preferably blinded), objective way in both cases and controls and/or failed to identify or appropriately control known confounders.

§§§ Split-sample validation is achieved by collecting all the information in a single tranche, then artificially dividing this into “derivation” and “validation” samples.

” “ An “Absolute SpPin” is a diagnostic finding whose Specificity is so high that a Positive result rules-in the diagnosis. An “Absolute SnNout” is a diagnostic finding whose Sensitivity is so high that a Negative result rules-out the diagnosis.

“¡”¡ Good, better, bad and worse refer to the comparisons between treatments in terms of their clinical risks and benefits.

” ” “ Good reference standards are independent of the test, and applied blindly or objectively to applied to all patients. Poor reference standards are haphazardly applied, but still independent of the test. Use of a non-independent reference standard (where the ‘test’ is included in the ‘reference’, or where the ‘testing’ affects the ‘reference’) implies a level 4 study.

” ” ” “ Better-value treatments are clearly as good but cheaper, or better at the same or reduced cost. Worse-value treatments are as good and more expensive, or worse and the equally or more expensive.

** Validating studies test the quality of a specific diagnostic test, based on prior evidence. An exploratory study collects information and trawls the data (e.g. using a regression analysis) to find which factors are ‘significant’.

*** By poor quality prognostic cohort study we mean one in which sampling was biased in favour of patients who already had the target outcome, or the measurement of outcomes was accomplished in <80% of study patients, or outcomes were determined in an unblinded, non-objective way, or there was no correction for confounding factors.

**** Good follow-up in a differential diagnosis study is >80%, with adequate time for alternative diagnoses to emerge (for example 1-6 months acute, 1 – 5 years chronic)
