# Supplementary material for: Treatment of chronic rhinosinusitis with nasal polyps (CRSwNP) with monoclonal antibodies (biologics): S2k guideline of the German Society of Oto-Rhino-Laryngology, Head and Neck Surgery (DGHNO-KHC), and the German College of General Practitioners and Family Physicians (DEGAM)
Source: HNO. 2023 Mar 20;71(4):256–63. [Article in German] doi: 10.1007/s00106-023-01273-2 (PMC10066152; doi:10.1007/s00106-023-01273-2)
Supplement: Supplementary file 2 [file 106_2023_1273_MOESM2_ESM.pdf]

Dokumentationsbogen

Indikationsstellung zur Therapie der schweren, unkontrollierten Form der CRScNP mit Biologika #

der S2k-Leitlinie ‚Therapie der chronischen Rhinosinusitis mit Polyposis nasi (CRScNP) mit monoklonalen Antikörpern (Biologika)' der Deutschen Gesellschaft für Hals-Nasen- Ohren-Heilkunde, Kopf- und Hals-Chirurgie (DGHNO-KHC) und der Deutschen Gesellschaft für Allgemeinmedizin und Familienmedizin (DEGAM)

Pfaar O, Beule AG, Laudien, Stuck BA, erweiterte Leitliniengruppe. HNO 2023; https://doi.org/10.1007/s00106-023-01273-2

**A. Voraussetzungen für In-Label Anwendung von Biologika bei CRScNP (alle sind zu erfüllen)**	
Schwere CRScNP	☐
Alter ≥ 18 Jahre	☐
Dauertherapie mit intranasalen Kortikosteroiden	☐
Keine ausreichende Kontrolle mit systemischen Kortikosteroiden und/oder chirurgischem Eingriff (Dupilumab/Mepolizumab), keine ausreichende Kontrolle durch intranasale Kortikosteroide (Omalizumab)	☐
**B. Indikationskriterien modifiziert/ in Anlehnung an Einschlusskriterien der Zulassungsstudien (optional)**	
Intranasale Steroide über 8 Wochen in der empfohlenen Dosierung	☐
NPS* ≥ 2 je Seite ** und in der Summe ≥ 5 (Grad rechts:____ Grad links:______)	☐
Rezidiv-Polyposis nach Eingriff (NNH-OP) in den letzten 10 Jahren	☐
Behandlung mit systemischen Kortikosteroiden i. d. letzten 2 Jahren (od. Kontraindikation)	☐
Fortbestehende Symptome (mind. 2, seit mind. 3 Monaten): Nasenatmungsbehinderung, anteriore Rhinorrhoe/post nasal drip, Gesichtsdruck/-schmerz, Riechminderung	☐
Gesamt-Beschwerden „Nasale Symptome" (VAS) >5 [Max. 10] (Wert:_______)	☐
SNOT-22*** Score ≥ 20 (Wert: ________)	☐
Bei Omalizumab: Gesamt-IgE 30-1500 IU/ml und dosierbar (nach Gewicht) (Wert/IU:____)	☐
**C. Unterstützende Dokumentation**	
**Komorbiditäten:**	
Erhebliches OP-Risiko (z. B. NYHA 3/4, nicht-pausierbare antikoagulative Therapie, Z. n. Schädelbasisrekonstruktion etc.)	☐
Erhöhtes Risiko für Anwendung systemischer Kortikosteroide (z. B. Diabetes mellitus, Ulcus duodeni, Thromboseneigung) [Diagnose(n):______________________________]	☐
Zeichen der Typ-2-Inflammation [☐ Asthma bronchiale (besonders ☐ allergisch / ☐ eosinophil), ☐ eosinophile Ösophagitis, ☐ Allergie (Art: ___________________________), ☐ atopische Dermatitis]	☐
Vorliegen eines Analgetika-Intoleranz-Syndroms (NERD, ASS-Intoleranz)	☐
**Weiterführende Informationen zu Vorbehandlungen:**	
Systemische Kortikosteroide über mindestens 5 Tage in einer Dosierung von mind. 0,5 mg/kg/KG – mindestens 2 x in den letzten 48 Monaten (Anzahl: ____)	☐
Auftreten einer Rezidiv-Polyposis weniger als 2 Jahre nach NNH-Operation: (____ Monate)	☐
Erfolglose adaptive ASS-Desaktivierung (z. B. aufgrund Nebenwirkungen oder fehlendem therapeutischen Effekt)	☐
**Weitere optionale Untersuchungsergebnisse:**	
Eosinophilie: ☐ im Blut (Wert:____(µl) / ☐ im histolog. Präparat (laut Pathologiebefund)	☐

© DGHNO-KHC/DEGAM 2022

# Weiterentwicklung des Dokumentationsbogens aus dem Positionspapier des AeDA und der DGHNO, doi: 10.1055/a-1197-0136

* NPS: 0: kein Polyp, 1: kleiner Polyp im mittleren Nasengang, der den unteren Rand der mittleren Mu- scheln nicht erreicht, 2: Polyp erreicht den unteren Rand der mittleren Muschel, 3: großer Polyp, der den unteren Rand der unteren Muschel erreicht oder Polyp medial der mittleren Muschel, 4: großer Polyp mit vollständiger Verlegung der Nasenhaupthöhle, Erreichung des Nasenbodens

** abweichend von den Zulassungskriterien halten die Autoren eine beidseitige Polyposis für eine Voraussetzung zur Anwendung.

*** Summenscore aus 22 Symptomkomplexen (Drang zum Schnäuzen, Verstopfte Nase, Niesen, Laufende Nase, Husten, Sekretfluss in den Rachen, dickes schleimiges Nasensekret, Druck auf dem Ohr, Schwindelgefühl, Ohrenschmerzen, Schmerzen oder Druckgefühl im Gesichtsbereich, verminderter Geruchs-/Geschmackssinn, Probleme beim Einschlafen, nächtliches Aufwachen, kein erholsamer Schlaf, Müdigkeit beim Aufwachen, Erschöpfung, Verminderte Produktivität, Verminderte Konzentrationsfähigkeit, Frustration/Unruhe/Reizbarkeit, Traurigkeit, Verlegenheit) mit Einzelscores von 0 bis 5 (keine, minimale, leichte oder schwache, mittelstarke, starke, schlimmstmögliche Beschwerden), nach doi: 10.1007/s00405-021-07019-6)

Abkürzungen: *ASS=Acetylsalicylsäure; CRScNP=Chronische Rhinosinusitis mit nasaler Polyposis; KG=Körpergewicht; NERD=Nonsteroidal antiinflammatory drug Exacerbated Respiratory Disease; NNH=Nasennebenhöhlen; NPS=Nasaler Polypenscore; NYHA=New York Heart Association; SNOT=Sino-Nasal Outcome Test; VAS=Visuelle Analogskala.*
