# Supplementary material for: Treatment of chronic rhinosinusitis with nasal polyps (CRSwNP) with monoclonal antibodies (biologics): S2k guideline of the German Society of Oto-Rhino-Laryngology, Head and Neck Surgery (DGHNO-KHC), and the German College of General Practitioners and Family Physicians (DEGAM)
Source: HNO. 2023 Mar 20;71(4):256–63. [Article in German] doi: 10.1007/s00106-023-01273-2 (PMC10066152; doi:10.1007/s00106-023-01273-2)
Supplement: Supplementary file 3 [file 106_2023_1273_MOESM3_ESM.pdf]

Leitlinien-Report der Aktualisierung des Kapitels „*Therapie der chronischen Rhinosinusitis mit Polyposis nasi (CRScNP) mit monoklonalen Antikörpern (Biologika)*“ (S2k) der S2k-Leitlinie „Rhinosinusitis“ 017-049 (HNO) und 053-012 (DEGAM)

Es handelt sich um eine Aktualisierung (Update) des Kapitels „***Therapie der chronischen Rhinosinusitis mit Polyposis nasi (CRScNP) mit monoklonalen Antikörpern (Biologika)***“ der S2k-Leitlinie „Rhinosinusitis“ der Deutschen Gesellschaft für Hals-, Nasen-Ohren-Heilkunde, Kopf- und Hals-Chirurgie (DGHNO-KHC) und der Deutschen Gesellschaft für Allgemeinmedizin und Familienmedizin (DEGAM). Die Aktualisierung erfolgte unter Beteiligung des Ärzteverbandes Deutscher Allergologen (ÄDA), des Berufsverbandes der Hals-Nasen-Ohrenärzte e.V. (BVHNO), der Deutschen Gesellschaft für Allergologie und Klinische Immunologie (DGAKI), der Deutschen Gesellschaft für Kinder- und Jugendmedizin (DGKJ) und Deutschen Gesellschaft für Pneumologie und Beatmungsmedizin (DGP).

1. Beteiligte Fachgesellschaften und Delegierte

Fachgesellschaft	Delegierter/e
Deutsche Gesellschaft für Hals-Nasen-Ohren-Heilkunde, Kopf- und Hals-Chirurgie (DGHNO)	A.G. Beule, Münster<br>M. Laudien, Kiel<br>O. Pfaar, Marburg<br>B.A. Stuck, Marburg
Deutsche Gesellschaft für Allgemeinmedizin und Familienmedizin (DEGAM)	U. Popert, Kassel
Ärzteverband Deutscher Allergologen (ÄDA)	L. Klimek, Wiesbaden
Deutscher Berufsverband der Hals-Nasen-Ohrenärzte e.V. (BVHNO)	C. Aletsee, Bad Kreuznach
Deutsche Gesellschaft für Allergologie und Klinische Immunologie (DGAKI)	M. Wagenmann, Düsseldorf
Deutsche Gesellschaft für Kinder- und Jugendmedizin (DGKJ)	M. Rose, Stuttgart
Deutsche Gesellschaft für Pneumologie und Beatmungsmedizin (DGP)	K. Milger-Kneidinger, München

AWMF-Registriernummer: 017-049 (HNO-KHC) und 053-012 (DEGAM)

2. Fertigstellung, Gültigkeit und geplante Überprüfung

2.1. Fertigstellung des Leitlinien (LL-) Kapitels

Die letzte (inhaltliche) Überarbeitung fand am 07. Juni 2022 statt.

2.2. Überprüfung des LL-Kapitels

Eine Überprüfung und evtl. Aktualisierung erfolgt im Rahmen der Gesamt-Aktualisierung der LL in 2023 durch die zwei Koordinatoren, Prof. Dr. med. Boris A. Stuck (DGHNO-KHC) und Dr. Popert (DEGAM) .

3. Geltungsbereich und Zweck

3.1. Begründung für die Auswahl des Leitlinienthemas

Monoklonale Antikörper (sog. Biologika) können bei der chronischen Rhinosinusitis mit Nasenpolypen (CRScNP, im englischen Sprachgebrauch CRSwNP) im Rahmen der Zulassung dieser Substanzen verordnet und angewendet werden. Allerdings ist die Verordnung auf die schwere CRScNP ohne Krankheitskontrolle begrenzt, wobei bestimmte Voraussetzungen zu beachten sind. Derzeit sind Dupilumab, Omalizumab und Mepolizumab zugelassen, und es findet sich in der Literatur entsprechende Evidenz für die Wirksamkeit und Sicherheit dieser Biologika. Es ist davon auszugehen, dass zukünftig weitere Biologika in dieser Indikation zugelassen werden.
Das vorliegende LL-Kapitel hat zum Ziel, angesichts der zunehmenden Evidenz zur Therapie mit diesen Substanzen bzw. der zunehmenden Zahl an Zulassungen unterschiedlicher Biologika zu einer qualitativ hochwertigen Versorgung von erwachsenen Patient*innen mit dieser Therapieform beizutragen.

3.2. Zielorientierung dieses Kapitels der Leitlinie

Das vorliegende Kapitel der LL hat zum Ziel, angesichts der zunehmenden Evidenz zur Therapie mit diesen Substanzen bzw. der zunehmenden Zahl an Zulassungen unterschiedlicher Biologika zu einer qualitativ hochwertigen Versorgung von erwachsenen Patient*innen mit dieser Therapieform beizutragen. Sie soll über die indikationsgerechte Diagnostik und Kriterien für den Einsatz von Biologika bei

CRScNP informieren, mit dem Ziel, die krankheitsbedingte Morbidität zu mindern. Darüber hinaus hat sie zum Ziel, vor dem Hintergrund der derzeit erheblichen Therapiekosten unter sozioökonomischen Gesichtspunkten einen optimalen Einsatz dieser Biologika zu gewährleisten.

3.3. Patientenzielgruppe

Patienten aller Altersgruppen beiderlei Geschlechts mit einer schweren Rhinosinusitis mit Nasenpolypen (CRScNP) ohne Krankheitskontrolle.

3.4. Versorgungsbereich

Dieses Kapitel der LL beschreibt die ambulante Versorgung im Rahmen der indikationsgerechten Anwendung der Biologika.

3.5. Anwenderziele/Adressaten

Die Leitlinie richtet sich an alle Ärzt*innen, die Patient*innen mit CRScNP im primär- und sekundärärztlichen Bereich behandeln. Primärärzt*innen verantworten die Erstbehandlung der Patient*innen, wobei diese Funktion in erster Linie unabhängig ist vom ärztlichen Fachgebiet bzw. der ärztlichen Spezialisierung. Sekundärärzt*innen sind in die Weiterbehandlung der Patient*innen eingebunden und behandeln die Patient*innen nach Überweisung durch den/die Primärarzt*in.

4. Zusammensetzung der Leitliniengruppe: Beteiligung von Interessensgruppen

4.1. Repräsentativität der Leitliniengruppe: Beteiligte Berufsgruppen

Das vorliegende LL-Kapitel wurde im Auftrag der Deutschen Gesellschaft für Hals-Nasen-Ohren-Heilkunde, Kopf- und Halschirurgie (DGHNO-KHC) und der Deutschen Gesellschaft für Allgemeinmedizin und Familienmedizin (DEGAM) als federführende Fachgesellschaften erstellt und finanziert. Es ist gemäß den methodischen Vorgaben der Arbeitsgemeinschaft der Wissenschaftlichen Medizinischen Fachgesellschaften (AWMF) auf einem S3-Niveau konzipiert worden in dem Bestreben, im Rahmen der kontinuierlichen Aktualisierung der Gesamtleitlinie diese schrittweise auf ein S3-

Niveau anzuheben. Als ersten Schritt erfolgte für dieses Kapitel eine systematische Evidenzaufbereitung im Sinne einer S3-Leitlinie.

Auf Vorstandsbeschluss der DGHNO-KHC wurden Prof. Dr. Boris A. Stuck und Prof. Dr. Oliver Pfaar und auf Vorstandsbeschluss der Deutschen Gesellschaft für Allgemeinmedizin und Familienmedizin (DEGAM) wurde Herr Dr. Popert im März 2020 beauftragt, die Koordination der Erstellung dieses Kapitels der LL zu übernehmen.
Es wurde unter Beteiligung des Ärzteverbandes Deutscher Allergologen (ÄDA), des Berufsverbandes der Hals-Nasen-Ohrenärzte e.V. (BVHNO), der Deutschen Gesellschaft für Allergologie und Klinische Immunologie (DGAKI), der Deutschen Gesellschaft für Kinder- und Jugendmedizin (DGKJ) und Deutschen Gesellschaft für Pneumologie und Beatmungsmedizin (DGP) erstellt. Die beteiligten Gesellschaften wurden im April 2020 eingeladen, Delegierte für die Konsensuskonferenzen zu benennen.

4.2. Interessenerklärungen und Umgang mit Interessenkonflikten

Interessenerklärungen wurden gemäß dem AWMF-Regelwerk rechtzeitig von allen Delegierten eingeholt und sie sind aufgeführt via https://register.awmf.org/assets/guidelines/017_D_G_f_Hals-Nasen-Ohrenheilkunde__Kopf-_und_Halschirurgie/017-049_und_053-012ikap_S2k_Rhinosinusitis_2022-11.pdf. Ebenfalls findet sich hier die von dem Ko-Koordinator Prof. Dr. med. Stuck und Herrn PD Dr. Sitter (neutraler externer Moderator, HS) durchgeführte Bewertung auf thematischen Bezug zur LL und die Einteilung bezüglich der Relevanz in gering, moderate und hohe Interessenkonflikte sowie die entsprechenden Konsequenzen (siehe auch „zweistufiges Abstimmungsverfahren“, Kapitel 5.2. und Kapitel 7.2).

4.3. Repräsentativität der Leitliniengruppe: Beteiligung von Patienten

Um auch die Meinung von Patienten/ Eltern von Patienten in den Überarbeitungsprozess einzubringen, wurde der Deutsche Allergie und Asthmabund (DAAB) angefragt, sich an der Erstellung zu beteiligen. Die Koordinatoren der LL wurden aber informiert, dass sich der DAAB nicht an der LL-Arbeit beteiligen möchte.

4.4. Repräsentativität der Leitliniengruppe: Absagen weiterer eingeladener Gesellschaften

Eingeladen für die Arbeit an diesem LL-Kapitel wurden zudem die Deutsche Gesellschaft für Hygiene und Mikrobiologie, die Deutsche Gesellschaft für Innere Medizin, der Deutsche Hausärzteverband, die Deutsche Röntgengesellschaft sowie die Deutsche Gesellschaft für Mund-Kiefer-Gesichtschirurgie e.V. (DGMKG), welche schriftlich informiert haben, nicht in die Überarbeitung eingebunden werden zu wollen.

5. Genauigkeit der Leitlinienentwicklung

Das vorliegende Kapitel ist entsprechend den methodischen Vorgaben zur Entwicklung von Leitlinien für Diagnostik und Therapie der in Deutschland etablierten Arbeitsgemeinschaft der Wissenschaftlichen Medizinischen Fachgesellschaften (AWMF) erstellt worden und entspricht nach dem Dreistufenkonzept der AWMF einem S2k-Niveau. Der gesamte, mit dem AWMF-Regelwerk konforme Prozess der Überarbeitung und Aktualisierung dieser Leitlinie ist durch Herrn PD Dr. Sitter begleitet und auf seine methodische Korrektheit überprüft worden.

5.1. Systematische Literaturbewertung, Evidenzlevel

Im Juni 2020 erfolgte die Formierung einer Arbeitsgruppe aus den Reihen der Delegierten der DGHNO-KHC mit dem Ziel, eine systematische Literaturrecherche für dieses Kapitel durchzuführen und auf dieser Grundlage einen Entwurf zu erstellen.
Für die systematische Literaturrecherche wurde die Leiterin der Zentralen Medizinischen Bibliothek der Philipps-Universität Marburg mit einbezogen. Als Suchbegriffe/Suchstrings für die Auswahl möglicher Literatur wurden diejenigen einer aktuellen Cochrane Metaanalyse (*Chong et al. Cochrane Database Syst Rev. 2020 Feb 27;2(2):CD013513. doi: 10.1002/14651858*) verwendet und die Literatur ab dem Jahr 2005 gesichtet. Die genutzten Suchbegriffe finden sich in Tabelle 1.

Die genutzten Datenbanken war Ovid MEDLINE(R) and Epub Ahead of Print, In-Process & Other Non-Indexed Citations, Daily and Versions(R) 1946 to August 20, 2020. Es lagen 1898 den Suchkriterien entsprechende Publikationen vor. Diese wurden von der Kerngruppe gesichtet und aufgrund ihres Bezuges zum Thema weiter

selektiert (Ausschluss von Dubletten, Ausschluss von Studien in anderen Indikationen als die CRSwNP, Ausschluss von Studien von Biologika, welche in Deutschland nicht zur Anwendung kommen), wobei 65 Einzelpublikationen übrigblieben. Diese wurden abschließend durch zwei Arbeitsgruppen (OP und BAS; AB und ML) erneut geprüft bezüglich der Relevanz für das eigentliche Thema (zulassungsrelevante/konfirmatorische Studien) und weiter eingegrenzt. Am Ende dieses Prozesses verblieben 17 Studien, welche auf der Basis der Evidenz-Bewertung nach der „*Checkliste der Bewertung von klinischen Studien der AWMF*“ und „*OXFORD-Einstufung: Evidenzlevel und Empfehlungsgrad*“ bewertet (Evidenz-Gradeinteilung nach den sog. Oxford-Kriterien von 2009, siehe Tabelle 2) und tabellarisch zusammengestellt wurden (siehe LL-Kapitel: „*Evidenztabelle*“). Bei erkannten Mängeln einer Studie oder erhöhtem Biasrisiko wurde eine Erniedrigung des Evidenzgrades vorgenommen.

Hieran anschließend erfolgte am 15.06.2021 eine Webkonferenz der Arbeitsgruppe, bei welcher der Aufbau des Kapitels diskutiert und die Einteilung der Beteiligten festgelegt wurde, um die entsprechenden Unterkapitel sowie den Dokumentationsbogen zu erarbeiten. In zwei weiteren Webkonferenzen am 11.11. und 02.12.2021 wurden die entsprechend vorbereiteten Teile des Kapitels sowie des Dokumentationsbogens von der Arbeitsgruppe diskutiert und modifiziert. Abgeschlossen wurde diese Arbeit durch die Arbeitsgruppe im Umlaufverfahren mit dem Ziel, den Entwurf der entsprechenden Dokumente rechtzeitig vor der eigentlichen Konsensuskonferenz allen beteiligten Delegierten zukommen zu lassen.

5.2. Strukturierte Konsensfindung: Verfahren und Durchführung

Die Konsensuskonferenz erfolgte am 20. April 2022 unter Moderation des AWMF-Beraters PD Dr. Helmut Sitter in Form einer Webkonferenz und wurde schriftlich einschließlich der Stimmenanteile protokolliert. Ferner erfolgte ein Videomitschnitt der kompletten Sitzung.
Nach vorheriger Zusendung des Kapitelentwurfes an alle Teilnehmer*innen wurde die Sitzung gemäß dem folgenden Ablaufschema durchgeführt:

1. Präsentation des Haupttextes sowie der zu konsentierenden Fazitsätze, Empfehlungen und des Dokumentationsbogens.

2. Stille Notiz: welcher Empfehlung/-Grad stimmen Sie nicht zu? Ergänzung, Alternative?
3. Registrierung der Stellungnahmen im Umlaufverfahren und Zusammenfassung von Kommentaren durch den Moderator.
4. Vorabstimmung über Diskussion der einzelnen Kommentare – Erstellung einer Rangfolge.
5. Debattieren/Diskussion der Diskussionspunkte.
6. Endgültige Abstimmung über jede Empfehlung und alle Alternativen.
7. Überarbeitung der Leitlinie gemäß den Beschlüssen.

Unter Moderation von Herrn PD Dr. Sitter konnte während dieser Konsensuskonferenz am 20. April 2022 das ganze Kapitel konsentiert und abgeschlossen werden.

Zu Beginn wurde die durch Prof. Dr. Stuck und PD Dr. Sitter getätigten Bewertungen der Interessenkonflikte (*Ausmaß der Interessenkonflikte für jede*n Stimmberechtigte*n (gering/moderat/hoch), Konsequenz für Abstimmungsverhalten,* siehe auch Kapitel 7.2) benannt. Die Abstimmung im weiteren Verlauf erfolgte in einem zweistufigen Prozess wie folgt: einmal mit allen Stimmberechtigten (Gesamtgruppe aller Delegierten mit Ausnahme des Ko-Koordinators Prof. Pfaar) und hiernach ein zweites Mal nur in der Untergruppe von Delegierten ohne oder mit geringem Interessenkonflikt. Sofern zwischen den Abstimmungsergebnissen ein relevanter Unterschied bestand, wurde das Abstimmungsergebnis der Untergruppe berücksichtigt. Bestand noch Diskussionsbedarf, wurde unter Moderation des externen neutralen Moderators PD Dr. Sitter diskutiert und ggf. erneut zweistufig abgestimmt. Der Ko-Koordinator Prof. Dr. Pfaar hat sich bei allen Abstimmungen inklusive Dokumentationsbogen (siehe unten) gemäß den Bewertungen der Interessenerklärungen enthalten und dies vor Beginn der Konsensuskonferenz mitgeteilt.

Bei den Empfehlungen wurde die Stärke des erreichten Konsenses im Rahmen der Abstimmungen beim nominalen Gruppenprozess in relativen Angaben versehen (Zustimmung). Gemäß AWMF-Regelwerk wurden die Konsensstärken wie folgt festgesetzt: >95%-100% starker Konsens, >75%-94% Konsens, mehrheitliche

Zustimmung >50%-74%, kein Konsens <50%. Alle Empfehlungen erhielten einen Konsens von mindestens 88 %.

Ferner konnten auch der Teil A und Teil B des Dokumentationsbogens komplett konsentiert und abgeschlossen werden. Im Anschluss an die Sitzung wurde den Delegierten die Möglichkeit eingeräumt, über mögliche Änderungen zum Teil C des Dokumentationsbogens in einem Delphiverfahren (E-Mail) zu informieren. Diese wurden durch Prof. Dr. Stuck und Prof. Dr. Pfaar entsprechend umgesetzt und erneut allen Beteiligten zur finalen Zustimmung gesendet. Diese erfolgte einvernehmlich bis einschließlich 12. Juni 2022.

5.3. Berücksichtigung von Nutzen, Nebenwirkungen-relevanten Outcomes

Ein zentraler Schwerpunkt der Leitlinie liegt in der Darstellung des (methodenspezifischen) Nutzens (Kapitel 8: „*Einsatz der Biologika in der Routineversorgung*“) sowie in der Darstellung des „*Dokumentationsbogens*“ (Anhang zur LL), welcher der Konsensusfindung zugrunde lag.

5.4. Formulierung der Empfehlung und Vergabe von Evidenzgraden und/oder Empfehlungsgraden

Die Empfehlungen wurden entsprechend dem von der AWMF empfohlenen Wording (*soll, sollte, kann*) formuliert und einzeln bei der Konsensuskonferenz unter AWMF-Moderation durch PD Dr. Sitter, abgestimmt. „Soll“ bedeutet eine starke Empfehlung, „Sollte“ eine mittelstarke Empfehlung und „Kann“ eine offene Empfehlung. Da es sich um eine S2k Leitlinie handelt, wurde die Graduierung der Empfehlungen sprachlich ausgedrückt und auf einen Empfehlungsgrad verzichtet.

6. Externe Begutachtung und Verabschiedung

6.1. **Pilottestung:** nicht durchgeführt.

6.2. **Externe Begutachtung:** nicht durchgeführt.

6.3. Verabschiedung durch die Vorstände der herausgebenden Fachgesellschaften/ Organisationen

Das Kapitel wurde durch die Vorstände der herausgebenden Fachgesellschaften/ Organisationen folgendermaßen verabschiedet.

Fachgesellschaft/ Organisation	Datum der Verabschiedung
DGHNO-KHC	08.08.2022
DEGAM	12.09.2022
ÄDA	25.07.2022
BVHNO	04.08.2022
DGAKI	26.08.2022
DGKJ	15.07.2022
DGP	22.07.2022

7. Redaktionelle Unabhängigkeit

7.1. Finanzierung der Leitlinie

Die Erstellung der Leitlinie, die Durchführung der Konsensuskonferenz unter Moderation durch den neutralen externen Moderator PD Dr. Sitter erfolgten durch Mittel der Deutschen Gesellschaft für Hals-Nasen-Ohrenheilkunde, Kopf- und Hals-Chirurgie e.V. (DGHNO-KHC). Die Leitlinienerstellung erfolgte in inhaltlicher Unabhängigkeit von der finanzierenden Organisation.

7.2. Darlegung von Interessen und Umgang mit Interessenkonflikten

Mandatsträger*innen und Koordinator*innen wurden fristgerecht vor der Konsensuskonferenz über die Geschäftsstelle der DGHNO per E-Mail aufgefordert, ihre Interessenerklärungen auf dem entsprechenden Portal der AWMF (https://interessenerklaerung-online.awmf.org/) zu hinterlegen. Die Beurteilung der Interessenerklärungen erfolgte durch Prof. Dr. Stuck und PD Dr. Sitter nach den AWMF-Empfehlungen zur Einteilung (**gering**: Tätigkeit als Berater*in und/oder Gutachter*in, bezahlte Vortrags-/oder Schulungstätigkeit, bezahlte Autor*innen-/oder Coautor*innenschaft; **moderat**: Mitarbeit in einem Wissenschaftlichen Beirat (advisory board), Forschungsvorhaben/ Durchführung klinischer Studien; wenn ein Bezug zur Leitlinie angegeben wurde; **hoch**: eigene finanzielle Beteiligung). Hohe Interessenkonflikte wurden nicht festgestellt, bei moderatem Interessenkonflikt erfolgte eine Stimmenthaltung im zweiten Abstimmungsverfahren (s.u.). Da ein Koordinator

(Prof. Dr. Pfaar) moderate Interessenkonflikte aufwies, wurde bei der Besetzung darauf geachtet, dass die beiden anderen Koordinatoren (Prof. Dr. Stuck, Dr. Popert) keine Interessenkonflikte hatten. Prof. Dr. Pfaar enthielt sich bei allen Empfehlungen und erklärte dies zu Beginn aller Sitzungen.
Vor Beginn der Online-Konsensuskonferenz erfolgte die Darlegung über das Ausmaß der Interessenkonflikte für jede*n Stimmberechtigte*n (gering/moderat/hoch) sowie evtl. Konsequenzen für die Stimmberechtigung der Delegierten durch PD Dr. Sitter (siehe auch Kapitel 5.2).
Die Interessenerklärungen sind tabellarisch – neben dem Leitlinienreport – auf der entsprechenden Internetseite der AWMF hinterlegt und dort abrufbar.

8. Verarbeitung und Implementierung

8.1. Konzept zur Verbreitung und Implementierung

Eine Veröffentlichung des Kapitels mit den ergänzenden Materialien soll über die AWMF (AWMF-Leitliniendatenbank) frei zugänglich im Internet erfolgen. Darüber hinaus sollen Publikationen in entsprechenden Fachzeitschriften erfolgen.

8.2. Unterstützende Materialien für die Anwendung der Leitlinien

keine konkrete Planung

8.3. Diskussion möglicher organisatorischer und/oder finanzieller Barrieren gegenüber der Anwendung der Leitlinienempfehlungen

keine konkrete Planung

8.4. Messgrößen für das Monitoring: Qualitätsziele, Qualitätsindikatoren

keine konkrete Planung

9. Gültigkeitsdauer und Aktualisierungsverfahren

9.1. Datum der letzten inhaltlichen Überarbeitung und Status

Die letzte (inhaltliche) Überarbeitung fand am 07. Juni 2022 statt.

9.2. Aktualisierungsverfahren

Eine Überprüfung und evtl. Aktualisierung dieses Kapitels erfolgt im Rahmen der Aktualisierung der Gesamt-Leitlinie „*Rhinosinusitis*“; verantwortlich für die Überarbeitung sind die beiden Koordinatoren Boris A. Stuck (DGHNO-KHC) und Uwe Popert (DEGAM).

Tabellen des Leitlinien-Reports

Tabelle 1: Recherecheterms für systematische Recherche

Ovid MEDLINE(R) and Epub Ahead of Print, In-Process & Other Non-Indexed Citations, Daily and Versions(R) 1946 to August 20, 2020

1 exp Sinusitis/
2 paranasal sinus diseases/ or rhinitis/ or rhinitis, atrophic/ or rhinitis, vasomotor/
3 exp Paranasal Sinuses/
4 (rhinosinusitis or nasosinusitis or pansinusitis or ethmoiditis or sphenoiditis).ab,ti.
5 (kartagener* adj3 syndrome*).ab,ti.
6 (inflamm* adj5 sinus*).ab,ti.
7 ((maxilla* or frontal*) adj3 sinus*).ab,ti.
8 1 or 2 or 3 or 4 or 5 or 6 or 7
9 exp chronic disease/
10 exp Recurrence/
11 (chronic or persis* or recur*).ab,ti.
12 9 or 10 or 11
13 8 and 12
14 CRSsNP.ab,ti.
15 ((sinusitis or rhinitis) adj3 (chronic or persis* or recur*)).ab,ti.
16 13 or 14 or 15
17 exp Nasal Polyps/
18 exp Nose/ or exp Nose Diseases/
19 exp Polyps/
20 18 and 19
21 ((nose or nasal or rhino* or rhinitis or sinus* or sinonasal) adj3 (papilloma* or polyp*)).ab,ti.
22 (rhinopolyp* or CRSwNP).ab,ti.
23 16 or 17 or 20 or 21 or 22
24 exp Antibodies, Monoclonal/
25 exp Antibodies, Anti-Idiotypic/
26 exp Immunoglobulin E/
27 exp INTERLEUKINS/
28 exp Receptors, Interleukin/
29 exp Biological Therapy/
30 exp Granulocyte-Macrophage Colony-Stimulating Factor/
31 exp Cytokines/
32 exp Etanercept/ or exp Alefacept/
33 (Antibod* adj3 monoclonal).ab,ti.
34 (Interleukin* or IgE or "immunoglobulin E" or Antiglobulin* or antiidiotyp*).ab,ti.
35 (anti adj3 (globulin* or idiotyp* or immunoglobulin* or M1 or CCR4 or "LFA 1" or "GATA 3" or OX40L)).ab,ti.
36 (ralokimumab or Adalimumab or Alemtuzumab or Bevacizumab or Certolizumab or Cetuximab or Denosumab or Ipilimumab or Natalizumab or Omalizumab or Palivizumab or Ranibizumab or Trastuzumab or stekinumab or mepolizumab or Nucala or SB240563 or "SB 240563" or dupilumab or REGN668 or AMG317 or "AMG 317" or AMG827 or "AMG 827" or DNAzyme or antiTSLP or CSL311 or "CSL 311" or "AMG 761" or AMG761 or "AMG 837" or KW0761 or "KW 0761" or "CSF 2" or "CSF GM").ab,ti.

37 (siliq or D2E7 or humira or campath or Lemtrada or avastin or cimzia or CDP870 or "CDP 870" or Erbitux or C225 or Xgeva or prolia or "AMG 162" or AMG162 or Yervoy or Tysabri or Antegren or Xolair or Synagis or Rhu-Fab or lucentis or Herceptin or stelara or CNTO or ASM8 or granulocyte-macrophage or GM-CSF or QGE031 or Raptiva or AK001 or "AK001").ab,ti.
38 ((B-cell or T-cell or Eosinophil or "mast cell" or stimulating) adj3 factor).ab,ti.
39 (CD23 or CD2 or CD11a or CD20 or CD25 opr CD252 or (receptor* adj3 apsilon)).ab,ti.
40 (CD adj3 ("23" or antigen* or "2" or 11a or "20" or "25" or "252")).ab,ti.
41 ((antigamma or "anti gamma") adj3 Antibod*).ab,ti.
42 (IgEid or "55700" or SCH55700 or CEP38072 or "CEP 38072" or cinqair or DCP835 or "DCP 835" or GM-CSF or TNF or TSLP or OX40L).ab,ti.
43 (IL adj3 ("5" or five or "4" or four or "13" or thirteen or "1" or one or "10" or ten or "11" or eleven or "12" or twelve or "15" or fifteen or "16" or sixteen or "17" or seventeen or "18" or eighteen or "2" or two or "23" or "twenty three" or "12" or twelve or "27" or "twenty seven" or "3" or three or "33" or "thirty three" or "6" or six or "7" or seven or "8" or eight or "9" or nine or 5R* or 1R1 or 4R* or 12p40 or IL-23p40 or 17A or 17RA or "22" or "twenty two" or "31" or "thirty one" or 31R)).ab,ti.
44 (IL5 or IL4 or IL13 or IL1 or IL10 or IL11 or IL12 or IL15 or IL15 or IL16 or IL17 or IL18 or IL2 or IL23 or IL12 or IL27 or IL3 or IL33 or IL6 or IL7 or IL8 or IL9 or IL22 or IL31).ab,ti.
45 (biologic or biologics or biotherap*).ab,ti.
46 (biologic* adj3 therap*).ab,ti.
47 (mAB or mepo or MDX or MEDI or siglec* or "lectin 8").ab,ti.
48 (SAR231893 or reslizumab or siglec8 or benralizumab or lebrikizumab or brodalumab or Tralokinumab or Quilizumab or Ligelizumab or Mogamulizumab or Efalizumab or Pitrakinra or Odulimomab or Mogamulizumabor or BCGF or binetrakin or "anti antibod*").ab,ti.
49 (siglec8 or TPI ASM8 or Rilonacept).rn.
50 (Canakinumab or Ilaris or Rilonacept or Arcalyst or Anakinra or Kineret or Antril or Altrakincept or Nuvance or Pascolizumab or SB 240683 or VAK694 or QBX258 or VAK 694 or VAK-694 or dectrekumab QAX-576 or QAX576 or QAX 576 or aerovant or AER-001 or AER001 or "AER 001" or BAY-16-9996 or BAY 16-9996 or Bosatria or Nucala or CDP 835 or CDP835 or CDP-835 or CINQAIR or CTx55700 or CTx 55700 or CTx-55700 or DCP 835 or DCP-835 or DCP835 or SCH5570 or SCH 5570 SCH-5570 or TRFK-5 or TRFK 5 or TRFK5 or BIW-8405* or BIW8405* or BIW 8405* or KHK 4563 or KHK-4563 or KHK4563 or Enokizumab or 7F3com-2H2 or Ustekinumab or Stelara or CNTO-1275 or CNTO1275 or CNTO 1275 or Anrukinzuma* or IMA-638 or PF-05230917 or GSK679586 or GSK-679586 or GDK 679586 or IMA026 or IMA-026 or "IMA 026" or IMA638 or IMA 638 MILR1444A or MILR 1444A or MILR-1444A or PRO-301444 or PRO301444 or PRO 301444 or RG-3637 or RG3637 or RG 3637 or RO-5490255 or RO5490255 or RO5490255 or TNX-650 or TNX650 or TNX 650 or RPC-4046 or ABT-308 or RPC4046 or ABT308 or RPC 4046 or ABT 308 or CAT-354 or CAT354 or CAT 354 or Secukinumab or Cosentyx or AIN-457 or KB-03303A or NVP-AIN 457 or AIN457 or KB03303A or NVPAIN457 or AIN 457 or KB03303A or NVP-AIN-457 or KHK-4827 or KHK4827 or KHK 4827 or fezakinumab* or ILV-094 or PF-5212367 or ILV094 or PF5212367 or "ILV094" or PF 5212367 or BMS-981164 or BMS981164 or BMS 981164 or Nemolizumab or CIM331 or CIM 331 or CIM-331 or Lenzilumab or KB003 or "KB 003" or KB-003 or ABT-D2E7 or D2E7 or LU 200134 or ABTD2E7 or LU200134 or ABT D2E7 or LU 200134 or Golimumab or Simponi or CNTO-148 or CNTO148 or CNTO 148 or Inflixima or cA2 or CenTNF or Remicade or TA-650 or TA650 or TA 650 or Etanercept or Enbrel or p75TNFR-Ig or rhu

TNFR-Fc or TNFR-Fc-p75 or TNR-001 or TNR001 or "TNR 001" or AMG-157 or MEDI-9929 or AMG157 or AMG 157 MEDI4212 or MEMP1972A or RG7449 or MEMP 1972A or RG 7449 or MEMP-1972A or RG-7449 or Mogamulizumab or KM8761 or Poteligeo or KM-8761 or KM 8761 or Alefacept or Amevive or "ASP 0485" or BG 9273 or BG 9712 or ASP0485 or BG9273 or BG9712 or ASP-0485 or BG-9273 or BG-9712 or Xanelim or Rituximab or Rituxan or Daclizumab or Zenapax or Oxeluma* or huMAb or OX40L or RG4930 or RO4989991 or RG4930 or RG-4930 or RO4989991 or RO-4989991 or Bertilimumab or Tezepeluma or Isunakinra or "Fusion Protein*" or cytokine*).ab,ti.

51	or/24-50
52	23 and 51
53	randomized controlled trial.pt.
54	controlled clinical trial.pt.
55	randomized.ab.
56	placebo.ab.
57	drug therapy.fs.
58	randomly.ab.
59	trial.ab.
60	groups.ab.
61	53 or 54 or 55 or 56 or 57 or 58 or 59 or 60
62	exp animals/ not humans.sh.
63	61 not 62
64	52 and 63
65	limit 64 to (yr="2005 -Current" and (english or german))
66	exp adult/
67	adult*.ab,ti.
68	66 or 67
69	65 and 68

Tabelle 2: Gradeinteilung der Evidenz (nach Bob Phillips, Chris Ball, Dave Sackett, Doug Badenoch, Sharon Straus, Brian Haynes, Martin Dawes (November 1998). Aktualisiert von Jeremy Howick im März 2009.)

Level	Therapy / Prevention, Aetiology / Harm	Prognosis	Diagnosis	Differential diagnosis / symptom prevalence study	Economic and decision analyses
1a	SR (with homogeneity*) of RCTs	SR (with homogeneity*) of inception cohort studies; CDR" validated in different populations	SR (with homogeneity*) of Level 1 diagnostic studies; CDR" with 1b studies from different clinical centres	SR (with homogeneity*) of prospective cohort studies	SR (with homogeneity*) of Level 1 economic studies
1b	Individual RCT (with narrow Confidence Interval"¡)	Individual inception cohort study with > 80% follow-up; CDR" validated in a single population	Validating** cohort study with good" " " reference standards; or CDR" tested within one clinical centre	Prospective cohort study with good follow-up****	Analysis based on clinically sensible costs or alternatives; systematic review(s) of the evidence; and including multi-way sensitivity analyses
1c	All or none§	All or none case-series	Absolute SpPins and SnNouts" "	All or none case-series	Absolute better-value or worse-value analyses " " " "
2a	SR (with homogeneity*) of cohort studies	SR (with homogeneity*) of either retrospective cohort studies or untreated control groups in RCTs	SR (with homogeneity*) of Level >2 diagnostic studies	SR (with homogeneity*) of 2b and better studies	SR (with homogeneity*) of Level >2 economic studies
2b	Individual cohort study (including low quality RCT; e.g., <80% follow-up)	Retrospective cohort study or follow-up of untreated control patients in an RCT; Derivation of CDR" or validated on	Exploratory** cohort study with good" " " reference standards; CDR" after derivation, or validated only on	Retrospective cohort study, or poor follow-up	Analysis based on clinically sensible costs or alternatives; limited review(s) of the evidence, or single

		split-sample§§§ only	split-sample§§§ or databases		studies; and including multi-way sensitivity analyses
2c	“Outcomes” Research; Ecological studies	“Outcomes” Research		Ecological studies	Audit or outcomes research
3a	SR (with homogeneity*) of case-control studies		SR (with homogeneity*) of 3b and better studies	SR (with homogeneity*) of 3b and better studies	SR (with homogeneity*) of 3b and better studies
3b	Individual Case-Control Study		Non-consecutive study; or without consistently applied reference standards	Non-consecutive cohort study, or very limited population	Analysis based on limited alternatives or costs, poor quality estimates of data, but including sensitivity analyses incorporating clinically sensible variations.
4	Case-series (and poor quality cohort and case-control studies§§)	Case-series (and poor quality prognostic cohort studies***)	Case-control study, poor or non-independent reference standard	Case-series or superseded reference standards	Analysis with no sensitivity analysis
5	Expert opinion without explicit critical appraisal, or based on physiology, bench research or “first principles”	Expert opinion without explicit critical appraisal, or based on physiology, bench research or “first principles”	Expert opinion without explicit critical appraisal, or based on physiology, bench research or “first principles”	Expert opinion without explicit critical appraisal, or based on physiology, bench research or “first principles”	Expert opinion without explicit critical appraisal, or based on economic theory or “first principles”

Notes

Users can add a minus-sign “-” to denote the level of that fails to provide a conclusive answer because:

- ***EITHER*** a single result with a wide Confidence Interval
- ***OR*** a Systematic Review with troublesome heterogeneity.

Such evidence is inconclusive, and therefore can only generate Grade D recommendations.

*	By homogeneity we mean a systematic review that is free of worrisome variations (heterogeneity) in the directions and degrees of results between individual studies. Not all systematic reviews with statistically significant heterogeneity need be worrisome, and not all worrisome heterogeneity need be statistically significant. As noted above, studies displaying worrisome heterogeneity should be tagged with a "-" at the end of their designated level.
"	Clinical Decision Rule. (These are algorithms or scoring systems that lead to a prognostic estimation or a diagnostic category.)
"¡	See note above for advice on how to understand, rate and use trials or other studies with wide confidence intervals.
§	Met when all patients died before the Rx became available, but some now survive on it; or when some patients died before the Rx became available, but none now die on it.
§§	By poor quality cohort study we mean one that failed to clearly define comparison groups and/or failed to measure exposures and outcomes in the same (preferably blinded), objective way in both exposed and non-exposed individuals and/or failed to identify or appropriately control known confounders and/or failed to carry out a sufficiently long and complete follow-up of patients. By poor quality case-control study we mean one that failed to clearly define comparison groups and/or failed to measure exposures and outcomes in the same (preferably blinded), objective way in both cases and controls and/or failed to identify or appropriately control known confounders.
§§§	Split-sample validation is achieved by collecting all the information in a single tranche, then artificially dividing this into "derivation" and "validation" samples.
" "	An "Absolute SpPin" is a diagnostic finding whose Specificity is so high that a Positive result rules-in the diagnosis. An "Absolute SnNout" is a diagnostic finding whose Sensitivity is so high that a Negative result rules-out the diagnosis.
"¡"¡	Good, better, bad and worse refer to the comparisons between treatments in terms of their clinical risks and benefits.
" " "	Good reference standards are independent of the test, and applied blindly or objectively to applied to all patients. Poor reference standards are haphazardly applied, but still independent of the test. Use of a non-independent reference standard (where the 'test' is included in the 'reference', or where the 'testing' affects the 'reference') implies a level 4 study.
" " " "	Better-value treatments are clearly as good but cheaper, or better at the same or reduced cost. Worse-value treatments are as good and more expensive, or worse and the equally or more expensive.
**	Validating studies test the quality of a specific diagnostic test, based on prior evidence. An exploratory study collects information and trawls the data (e.g. using a regression analysis) to find which factors are 'significant'.
***	By poor quality prognostic cohort study we mean one in which sampling was biased in favour of patients who already had the target outcome, or the measurement of outcomes was accomplished in <80% of study patients, or outcomes were determined

in an unblinded, non-objective way, or there was no correction for confounding factors.

**** Good follow-up in a differential diagnosis study is >80%, with adequate time for alternative diagnoses to emerge (for example 1-6 months acute, 1 – 5 years chronic)
